# Dynamic Flow-Assisted Nanoarchitectonics

**DOI:** 10.1021/acsami.5c03820

**Published:** 2025-04-21

**Authors:** Katsuhiko Ariga, Shuta Fujioka, Yu Yamashita

**Affiliations:** † Research Center for Materials Nanoarchitectonics, National Institute for Materials Science (NIMS), 1-1 Namiki, Tsukuba 305-0044, Japan; ‡ Graduate School of Frontier Sciences, The University of Tokyo, 5-1-5 Kashiwanoha, Kashiwa, Chiba 277-8561, Japan

**Keywords:** device, interface, Langmuir−Blodgett method, layer-by-layer assembly, nanoarchitectonics, natural flow, organic semiconductor

## Abstract

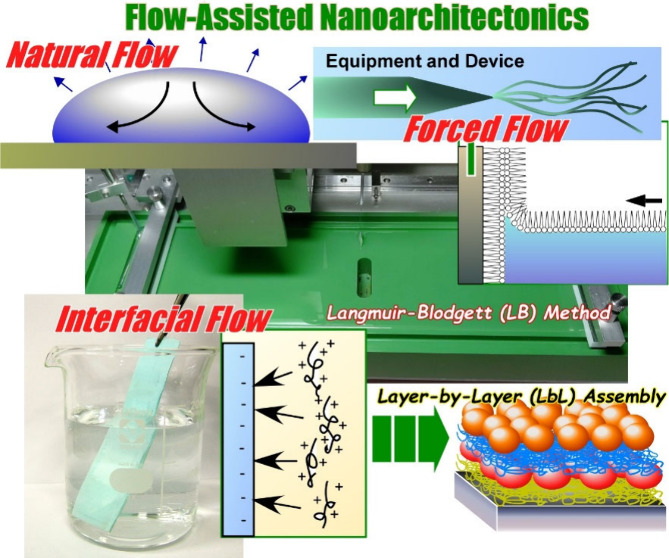

The solution to societal problems such as energy, environmental, and biomedical issues lies in the development of functional material systems with the capacity to address these problems. In the course of human development, we are entering a new era in which nanostructure control is considered in the major development of functional materials. The new concept of nanoarchitectonics is particularly significant in this regard, as it comprehensively promotes further development of nanotechnology and its fusion with materials chemistry. The integration of nanoscale phenomena and macroscopic actions is imperative for practical production of functional materials with nanoscale structural precision. This review focuses on dynamic flow-assisted nanoarchitectonics, wherein we explore the organization and control of functional structures by external mechanical stimuli, predominantly fluid flow. The review then proceeds to select some examples and divide them into categories for the purpose of discussion: structural organization by (i) natural flow, (ii) flow or stress created with artificial equipment or devices (forced flow), and (iii) flow at a specific field, namely interfaces, that is, layer-by-layer (LbL) assembly and the LB method. The final perspective section discusses the future research directions and requirements for dynamic flow-assisted nanoarchitectonics. The meaningful and effective use of nanotechnology and nanoarchitectonics in materials science is set to be a major area of focus in the future, and dynamic flow-assisted nanoarchitectonics is poised to play a significant role in achieving this objective.

## 1 Introduction

The key to solving societal problems such as energy,^1−4^ environment,^5−8^ and biomedical^9−12^ issues is the development of functional material systems capable of solving these problems. In the history of mankind, functional materials have been developed through the creation of substances and the shaping and processing of these substances. In other words, both the science of material creation and the technology of their processing have long helped society as an essential approach. In terms of material creation, developments in various fields of chemistry^13−16^ have contributed to these developments especially since the 20th century. In the midst of these developments, it has become clear over the past half century that controlling nanostructures is important for improving functionality.^17−20^ Even within the same material, functionality can be significantly improved or new functions can emerge, depending on the rational design of its internal nanostructures.^21−24^ During the long history of human development, we are entering a new era where nanostructure control is being considered in the major development of functional materials.

Nanotechnology, which has flourished since the second half of the 20th century,^25,26^ has contributed greatly to the development of micro and nanofabrication techniques that support the control of nanostructures and the understanding of nanosciences. It has become possible to observe and evaluate the structures of atoms, molecules, and nanomaterials,^27−30^ manipulate them^31−34^ and evaluate their physical properties in nanoenvironments.^35−38^ The development of microfabrication and nanofabrication in device fabrication has also contributed greatly.^39−42^ Various developments in materials chemistry have been made to address these issues. For example, template synthesis in inorganic and organic chemistry,^43−46^ molecular recognition and assembly in supramolecular chemistry,^47−50^ metal–organic frameworks (MOFs) in coordination chemistry,^51−54^ covalent organic frameworks (COFs) in polymer chemistry,^55−58^ self-assembled monolayers (SAMs),^59−61^ the Langmuir–Blodgett (LB) method,^62−64^ and layer-by-layer (LbL) assembly^65−67^ in interfacial chemistry have advanced in parallel with developments in nanotechnology. Other contributions include the creation of hybrids and composites of multiple materials,^68−70^ the manipulation of atoms and molecules to control material structures,^71−73^ nano- and microfabrication,^74−76^ and even the synthesis of materials through biochemical processes.^77−79^ As essential paradigms on how to assemble nanounits to functional materials, basic concepts self-assembly^80−82^ and self-organization^83−85^ have a central importance. In addition, emerging concepts such as instructed assembly,^86−88^ directed assembly,^89−91^ localized assembly,^92,93^ and biomaterial assembly upon liquid phase separation^94,95^ can lead to the formation of asymmetric, heterogeneous and hierarchical structures with greater spatial and temporal precision. They can be integrated into the new concept of nanoarchitectonics,^96−98^ which is an unified concept for construction of functional materials from the nanoscale units.^99−101^ This could become a concept that comprehensively promotes the further development of nanotechnology and its fusion with materials chemistry.^102−104^

In addition to these scientific basics, process integration between nanoscale phenomena and macroscopic actions is necessary for practical production of functional materials with nanoscale structural precision. In this review we consider the control of nanostructures by macroscopic external forces from a technical and engineering perspective. Macroscopic processing technology and nanoscale organization control are fused into macro- and nanoprocess integrations. This is a global issue of how to combine traditional technologies that have been used since ancient times with nanotechnology that we have developed in recent years. Similar attempts have been made in many scientific fields including mechanochemistry,^105−107^ for example. This technology controls chemical reactions and molecular organization by applying external forces. In addition, structural control by various external stimuli such as light irradiation, even if not mechanical, can be included in this category.^108−110^ It is also possible to consider structural changes in biorelated functional organisms due to external stimuli.^111−113^

Among these various phenomena, when considering the control of nanomaterial structures and molecular structures by external forces and energy, it is well-known that there is a large difference between artificial and biological systems.^114^ In artificial systems, light irradiation and mechanical stimuli often induce fairly clear structural changes using large input energy. In contrast, in biological systems, subtle but sophisticated functions are controlled by molecular motion and conformational changes using weak forces and energies that compete with thermal fluctuations.^115,116^ In other words, living organisms perform a more delicate process integration between nano and macro. The latter area has not been fully explored in artificial systems. An example of a weak and delicate force in an artificial system is structural control by fluid flow, such as the compression of a monolayer at the air–water interface.^117^ It has been shown that the actions and flows on a liquid can control molecular conformations, such as the dihedral angle of binaphthyl,^118^ and drive molecular machines.^119−121^ Although the magnitude of the force acting on the liquid flow varies, it has the potential to apply minute perturbations that can at least control the structure of delicate molecules and materials.

**Figure 1:**
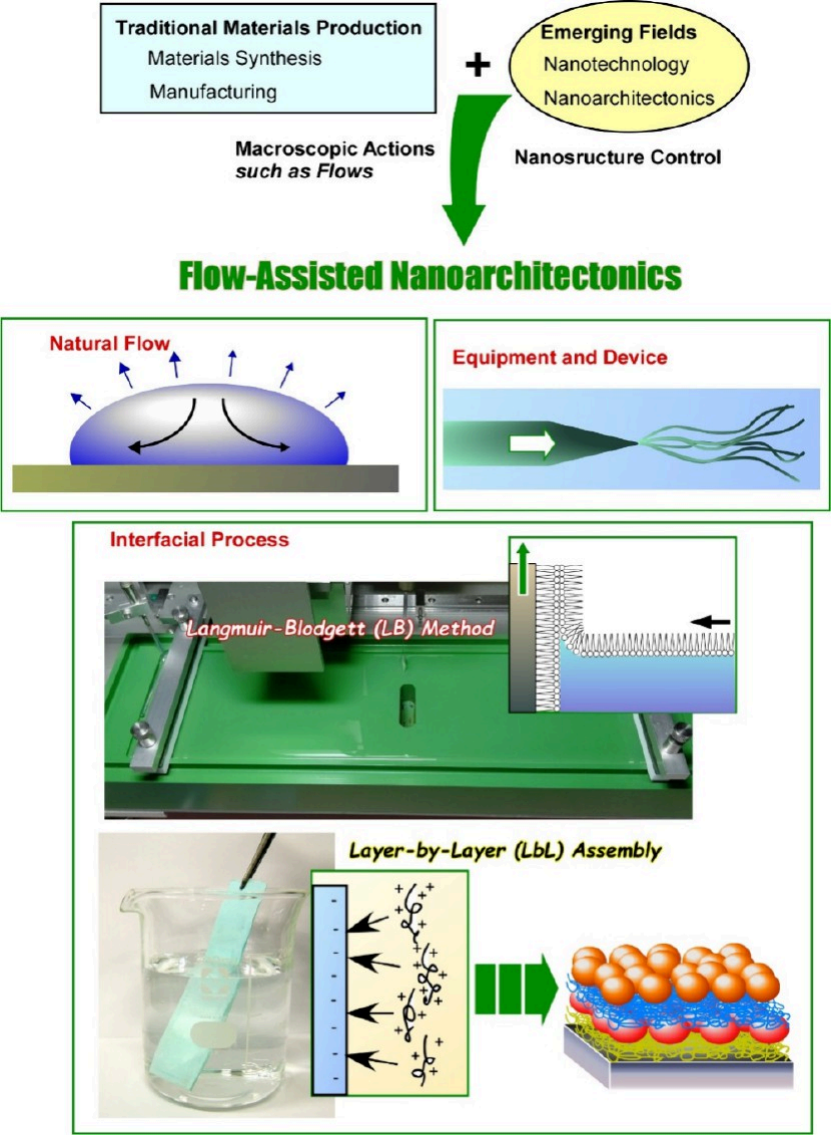
Outline of this review article for flow-assisted nanoarchitectonics mainly including (i) structural organization by natural flow, (ii) structural organization by artificial flow or stress created by equipment or devices, and (iii) structural organization by flow at interfaces (LB method and LbL assembly).

Based on this background, this review focuses on dynamic flow-assisted nanoarchitectonics, in which we discuss the organization and control of functional structures by external mechanical stimuli, mainly fluid flow (Figure 1). There are many examples of flow-induced structure formation, and thus, it is difficult to include everything in one small review article. Therefore, we classified several examples according to the method and media used to generate the flow. In this review, we selected some examples and divided them into (i) structural organization by natural flow, (ii) structural organization by flow or stress created by artificial equipment or devices (forced flow), and (iii) structural organization by flow in a specific field, namely interfaces. In the last examples of research at interfaces, we consider examples where a flow element is added to thin film formation by LbL assembly and the LB method, which are representative methods for structure formation. Finally, we examine specific targets, organic semiconductor thin films as case studies. Considering these facts, future research directions and requirements for dynamic flow-assisted nanoarchitectonics are discussed in the final section. This review will especially impress unavoidable contributions of dynamic flow systems to materials nanoarchitectonics, which are systematically categorized by representative flow types such as natural flows, forced flows, and interfacial flows.

## 2 Organization by Natural Flows

In the first section, we present some examples of organizations using natural flows that can serve as a basis for flow-assisted nanoarchitectonics. Natural phenomena, including solvent evaporation, can induce flows such as natural convection, Marangoni flow, and capillary flow, which can lead to the organization of functional materials contained in solutions as well as an increase in the material concentration and ordering at the meniscus.

**Figure 2:**
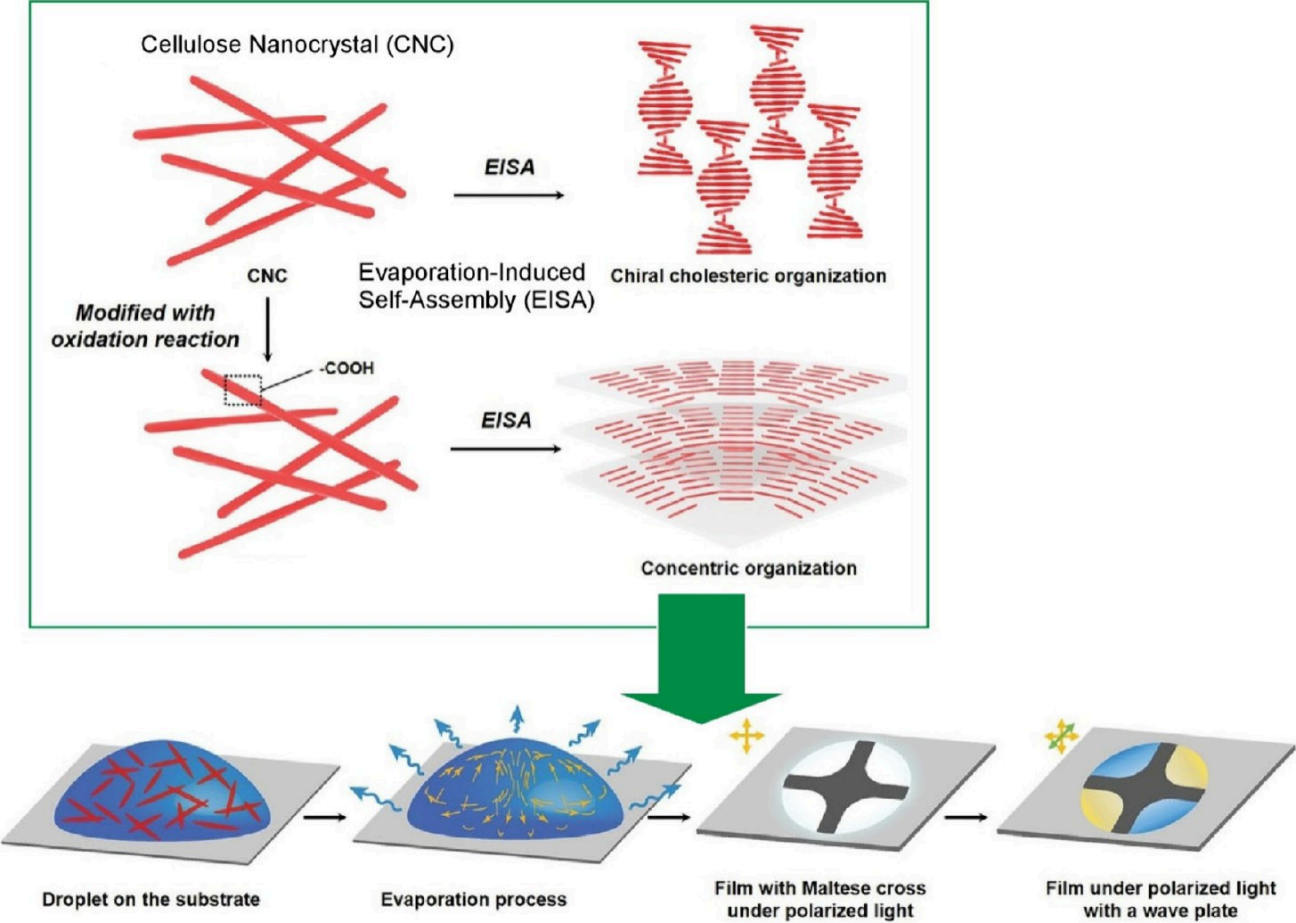
Approaches to assemble cellulose nanocrystals in a concentric manner as attributed to the circulating flow within the droplets, which results from the synergistic and competitive interaction of the Marangoni flow and the capillary effect during the evaporation. Adapted with permission from ref (125). Copyright 2021 Wiley-VCH.

Different functions can be achieved by orienting and assembling nanomaterials. For example, the controlled organization of cellulose nanocrystals is expected to be an advanced photonic material with new functions.^122−124^ This is important for various applications such as optics, sensing, and security. Instead of the traditional cholesteric assembly method, Ye and co-workers reported an approach for assembling cellulose nanocrystals in a concentric manner using capillary flow and the Marangoni effect (Figure 2).^125^ According to their method, simple evaporation of a solution of carboxyl-functionalized cellulose leads to a concentric assembly of nanocrystals like negative spherulites. This phenomenon is attributed to the circulating flow within the droplets, which results from the synergistic and competitive interaction of the Marangoni flow and capillary effect during the evaporation of the cellulose nanocrystal droplets. By grafting carboxyl functional groups onto the cellulose nanocrystals, a surface tension gradient is enabled to control the organization behavior of the cellulose nanocrystals during droplet evaporation. The distinct flow patterns created by a combination of the temperature-dependent Marangoni effect and capillary action allow us to manipulate the unique collection modes and corresponding optical properties by simply changing the evaporation temperature. The concentric superstructure gives them special optical properties that exhibit Maltese cross-optical properties under linearly polarized light. This feature can be switched on and off. This organizational structure is expected to have various functions such as information encryption, anticounterfeiting technology, and encrypted inkjet printing. Combined with 3D inkjet printing technology, any security information such as QR codes can be encoded. Multipurpose applications are expected for banknotes, pharmaceuticals, food, clothing, and microelectronics.

**Figure 3:**
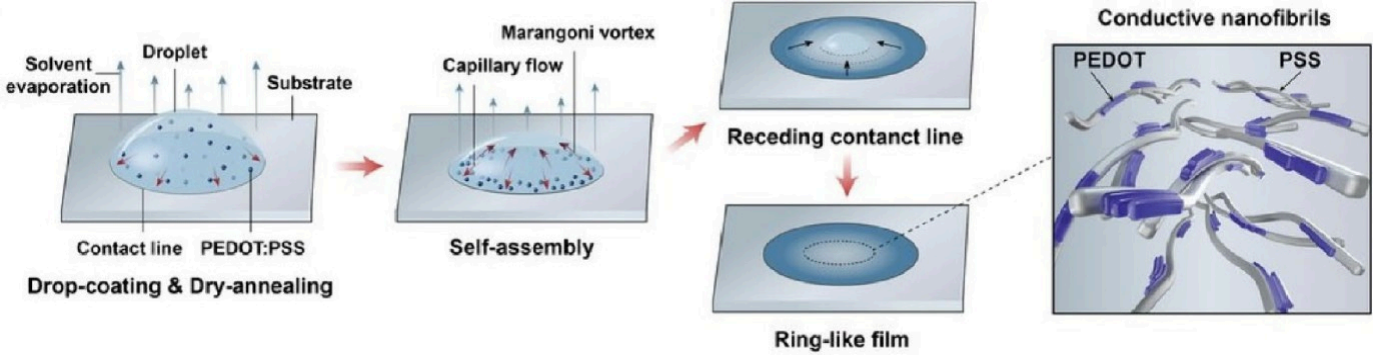
Scalable fabrication of conductive PEDOT:PSS inks via metastable liquid–liquid contact as a ring-shaped film formed by combination between the coffee ring effect and Marangoni vortices during droplet evaporation. Reprinted with permission from ref (129). Copyright 2023 Wiley-VCH.

The assembly of materials with electrical properties leads to the development of materials for electrical and electronic applications. For example, the creation of functionally assembled structures from high-performance polymers such as poly(3,4-ethylenedioxythiophene):poly(styrenesulfonate) (PEDOT:PSS) is increasingly required for flexible electronic devices.^126−128^ Shan, Du and co-workers reported the scalable fabrication of conductive PEDOT:PSS inks via metastable liquid–liquid contact (Figure 3).^129^ In this method, a ring-shaped film is formed by the combination of the coffee ring effect and Marangoni vortices during droplet evaporation. This is a simple solution processing method to convert an aqueous PEDOT:PSS solution into an ethylene glycol-based polymer solution with a low PSS content. A certain amount of PEDOT:PSS aqueous solution is dropped slowly and gently onto the ethylene glycol surface to maintain a layered state. The dopant ethylene glycol at the bottom gradually penetrates into the aqueous solution at the top. Drop coating leads to the self-assembly of PEDOT:PSS particles via capillary flow. Initially, the outward capillary flow dominates the flow field and carries the polymer particles to the edges. As evaporation continues, the decrease in the thickness of the droplet meniscus and the rapid regression of the contact line weaken the stacking of the polymer particles and align the polymers along the contact line. The concentration of the PEDOT:PSS polymer remaining at the center is much lower than that at the edges. As a result, the edge regions are patterned with concentric bands with height gradients. This allows for a ring-like morphology and a preferentially interconnected network of PEDOT:PSS nanofibrils, resulting in a high electrical conductivity and excellent optical transparency of the film. It is useful as an electrode for touch sensors with gradient pressure sensitivity. It has a wide range of applications in capacitive pressure sensors, as an electrode designed to mimic the gradient pressure distribution of a fingertip when pressed. This method may be a promising self-organized integration method for organic semiconductor films, not limited to PEDOT:PSS. It is expected to have applications in flexible electronics, bioelectronics, and photovoltaic devices.

**Figure 4:**
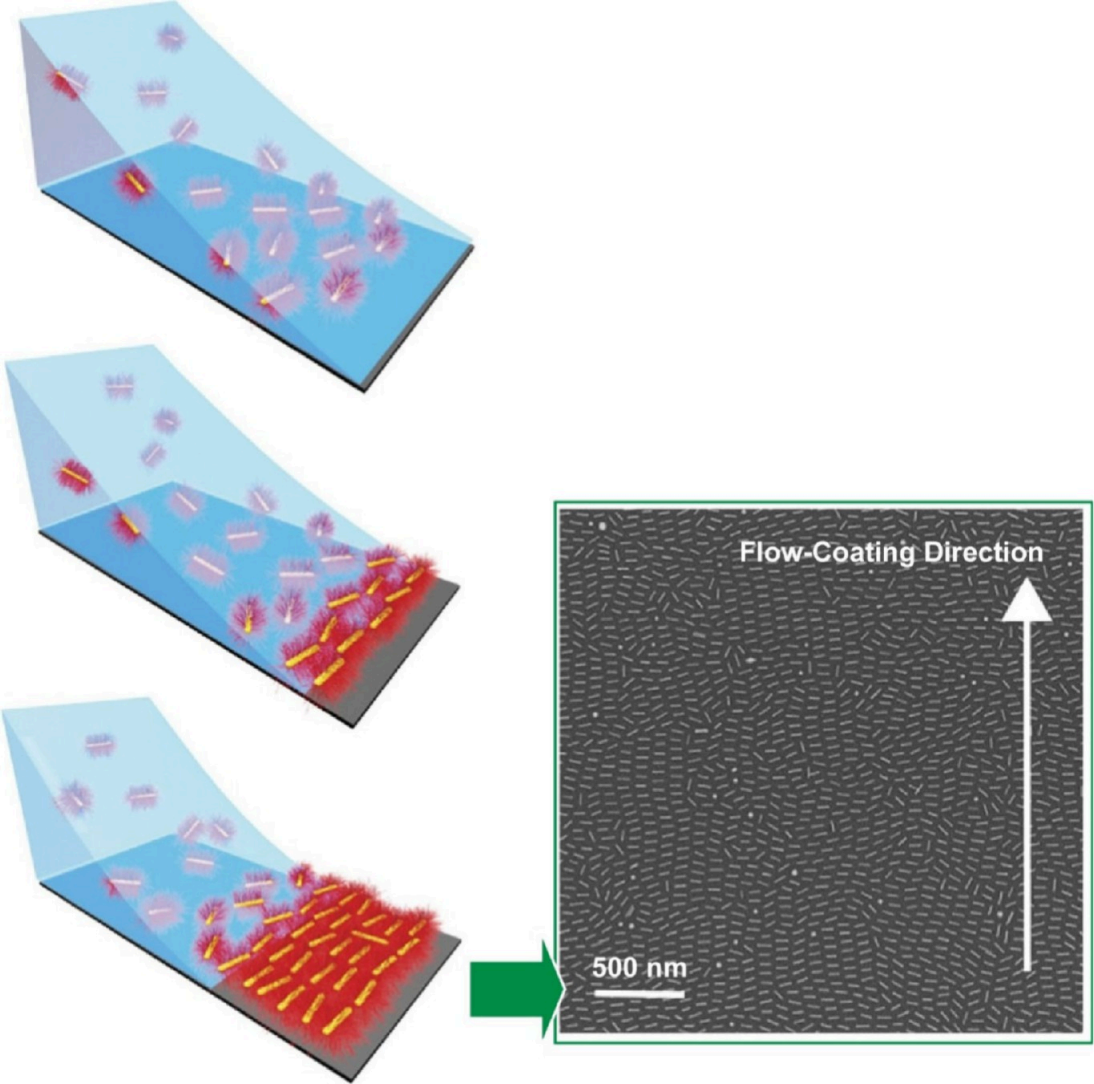
Fabrication of robust, free-standing mono- and bilayer films of polystyrene-grafted gold nanorods on solid substrates using flow coating. Adapted with permission from ref (132). Copyright 2021 American Chemical Society.

The organization of shape-defined nanomaterials, such as gold nanorods, is also key to the manufacture of functional materials.^130,131^ For example, solution-based printing techniques of anisotropic nanostructures form the basis of many emerging technologies, including energy storage devices, photonic elements and sensors. The rapid fabrication of large-area assemblies with simultaneous control over the thickness, nanomaterial spacing, surface roughness, and global and local orientational order is critical. Vaia and co-workers reported the fabrication of robust, free-standing mono- and bilayer films of polystyrene-grafted gold nanorods on solid substrates using flow coating (Figure 4).^132^ The combination of large capillary flows and preferential alignment of the nanorods parallel to the edge of the meniscus produces globally aligned films with a smectic-like order when operated in the evaporation mode. In particular, slower velocities in the evaporation regime result in global alignment of the polystyrene-grafted gold nanorods. Solvent evaporation at the meniscus induces capillary flow, causing the polystyrene-grafted gold nanorods to flow toward the edge of the contact line. As a result, the number density of polystyrene-grafted gold nanorods near the meniscus becomes much higher than that of the bulk solution, creating a concentration gradient. This sudden increase in the concentration induces a local transition from isotropic to aligned. The distance between rods can be finely controlled by the processing speed and surface energy of the substrate. As a result, the optical extinction of a specific polystyrene-grafted gold nanorod film can be tuned by controlling the plasmonic coupling between adjacent nanorods. This means that fundamental process-structure relationships can be used to tune the distance- and orientation-dependent plasmonic coupling of large gold nanorod arrays and systematically vary the optical properties of the films. The fundamental principles established in this work may have further applications, such as the use of polymer-grafted semiconducting nanorods to fabricate globally aligned thin films for polarized light-emitting displays, and may also be broadly applicable to the construction of other anisotropic nanostructured systems.

**Figure 5:**
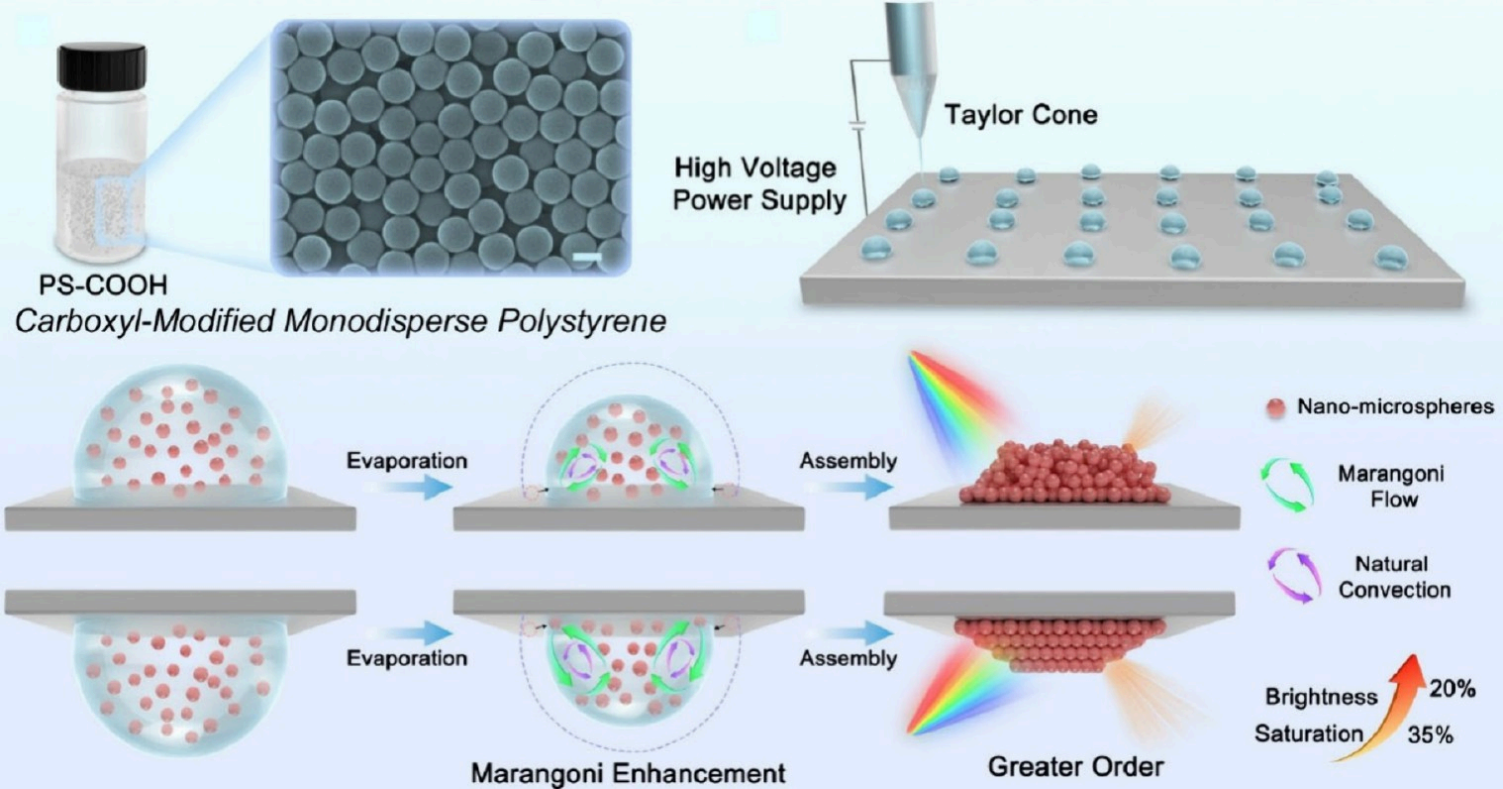
Formation of structural color materials through synchronizing natural convection and Marangoni flow in pendant drops. Reprinted with permission from ref (136). Copyright 2024 American Chemical Society.

The oriented assembly of nanomaterials and colloidal particles produces a function called structural color.^133−135^ Structural color is known for its persistent vividness and has been widely developed and applied in the fields of display and anticounterfeiting. To create materials that exhibit structural color, Feng and co-workers propose a pendant evaporation self-assembly method as shown in Figure 5.^136^ This method focuses on exploiting the synergistic enhancement of the flow field induced by pendant evaporation via natural convection and the Marangoni flow. The self-assembly process extends the dynamics and duration of colloidal nanoparticles, thereby improving the order of the colloidal photonic crystals. In this study, they demonstrate experimentally and theoretically, using a particle image velocimetry system and finite element simulations, that the synergistic coupling of natural convection and Marangoni flow is the main driving force for the self-assembly of nanoparticles. The optimized strategy of pendant evaporation-induced structural coloring is simple to operate and has the potential for industrial production. It is expected to further expand the technical applications in important areas such as displays and anticounterfeiting measures.

**Figure 6:**
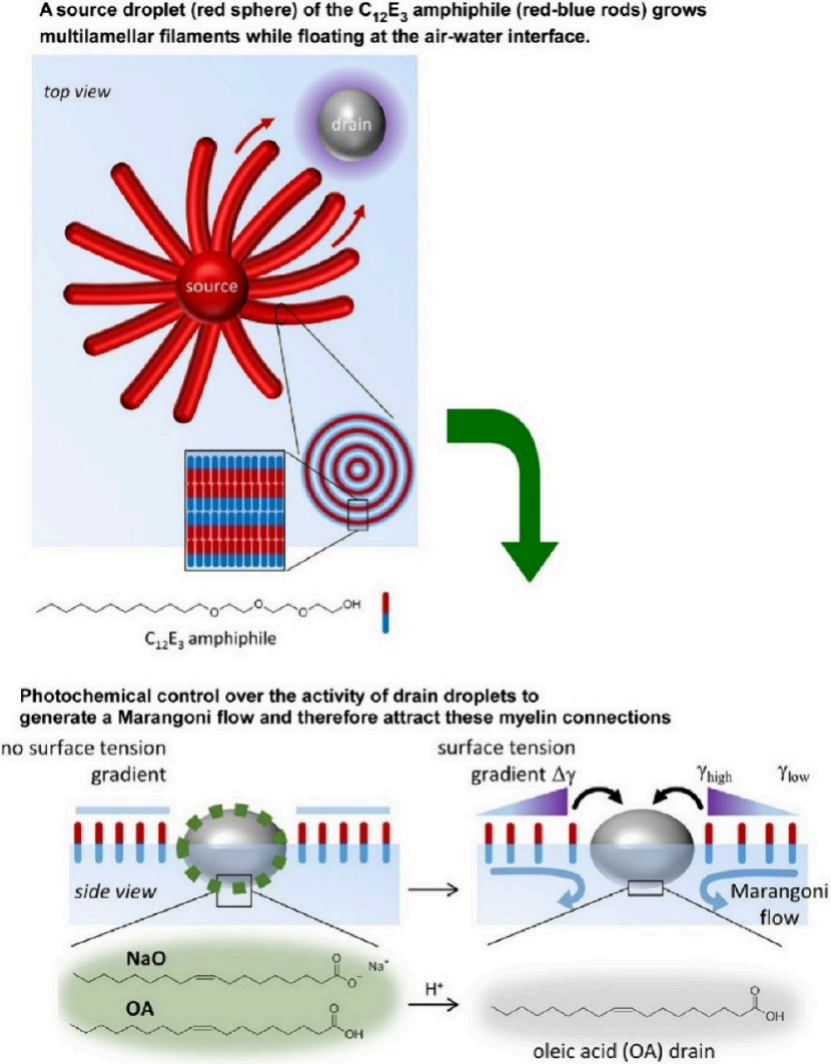
A method to create droplet-to-droplet chemical signaling structures using a combination of surfactants, self-assembly and photochemistry, where myelin-like wire structures were formed using the surfactant triethylene glycol monododecyl ether through photocontrolled Marangoni flow. Reproduced under terms of the CC-BY license from ref (137), 2024 American Chemical Society.

Softer structures with complex shapes can also be organized using flow. Inspired by signaling between the dispersed units of the slime mold Physarum polycephalum, Korevaar and co-workers report a method to create droplet-to-droplet chemical signaling structures using a combination of surfactants, self-assembly and photochemistry (Figure 6).^137^ Myelin-like wire structures were formed using the surfactant triethylene glycol monododecyl ether. Myelin is originally an extended, sponge-like structure of the oligodendrocyte cell membrane. They used photocontrolled Marangoni flow. They photochemically controlled the activity of the droplets to generate a Marangoni flow and attract myelin junctions. In particular, they developed a method to direct the trajectory of these myelins to selected photoactivated droplets upon UV irradiation. Localized UV irradiation attracts myelin and forms self-organizing and reconfigurable connections between the droplets. It can establish chemical communication through myelin filaments, the trajectories of which can be controlled by light from the source to the drain droplets. Although the structure formation is elegant, the materials used are based on molecular building blocks that are simple, readily available, and amenable to chemical modifications. They can provide design principles that can be transferred to spatiotemporally controlled droplet networks. Droplets may be attractive building blocks for the organization of dynamic matter into adaptive structures. Communication between droplets opens up untapped opportunities in various fields, including sensing, optics, protocells, computing or adaptive materials.

The above examples show how flows, which can occur as a result of spontaneous processes or stimuli, can naturally form material organizations with greater structural information. These examples demonstrate that common phenomena such as naturally occurring flows can produce more advanced structures. Although not mentioned in this review article, it is widely known that natural phenomena such as liquid phase separation can promote the organization of various substances and biological materials and play important biological roles.^138,139^ Skillful design of the phenomena that give rise to such dynamic, localized assemblies is likely to be important in the creation of advanced functional structures.

## 3 Organization by Forced Flows Using Devices

In the previous section, we provided examples of the organization of matter using naturally occurring flows. These flows, which can occur anywhere, can lead to sophisticated organizational structures. It is not difficult to imagine that we can go further and create artificial forced flows using tools and equipment, which could lead to even more sophisticated developments. In this section, we present examples of material organization through forced flows.

**Figure 7:**
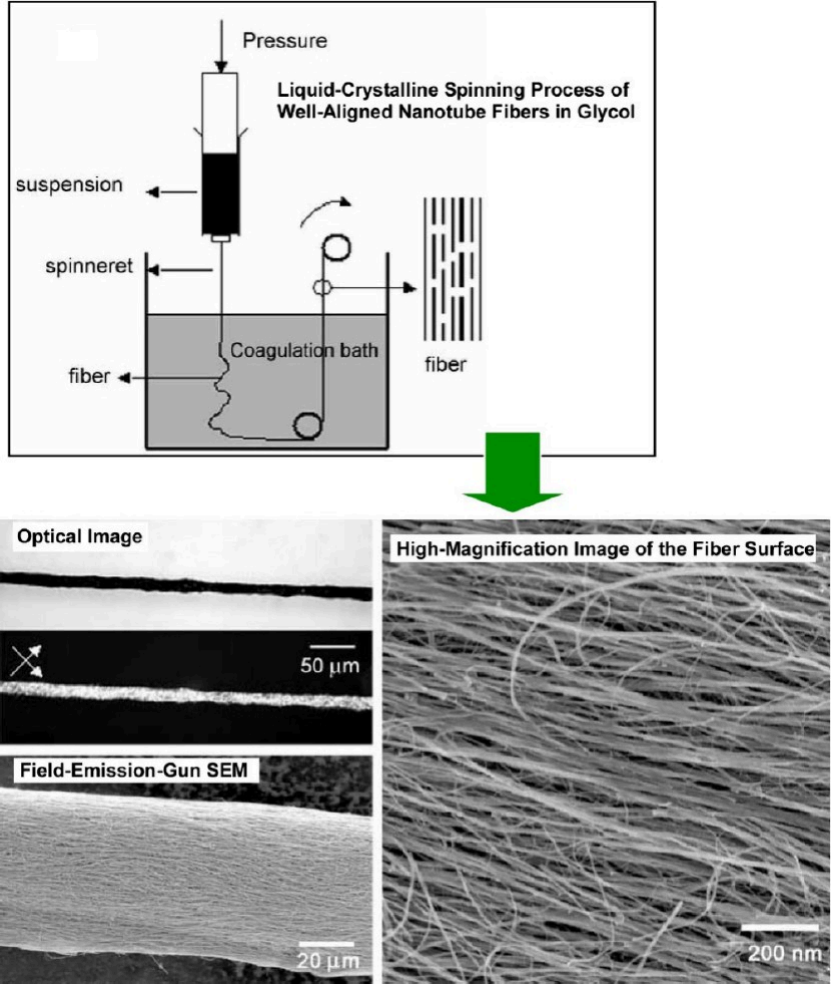
A coagulation process for spinning multiwalled carbon nanotubes from a liquid crystalline ethylene glycol dispersion. Adapted with permission from ref (140). Copyright 2008 Wiley-VCH.

Various techniques used in the manufacture of materials can be applied to organize nanostructures using flow and motion. Spinning is a typical example of this phenomenon. During spinning, the raw materials are liquefied and extruded into fibers through a nozzle. For example, Windle and co-workers reported a simple process for spinning multiwalled carbon nanotube fibers directly from a lyotropic liquid crystal phase (Figure 7).^140^ This is a coagulation process for spinning multiwalled carbon nanotubes from a liquid crystalline ethylene glycol dispersion. The nanotubes were ultrasonically dispersed in ethylene glycol. The dispersions changed from isotropic to biphasic to nematic phases with increasing concentrations. These dispersions were extruded as fibers in a diethyl ether bath. The domains of the nematic nanotube dispersion were shear-aligned as they passed through the needle. Upon entering the ether phase, ethylene glycol rapidly diffused out of the extruded nanotube fibers into the ether, and the ether diffused back into the fibers. At this stage, the fibers had a circular cross-section. Owing to the applied shear forces and formation of liquid crystal phase, the nanotubes were highly aligned within the fibers. The doped nanotubes were spun into fibers with a higher packing density, resulting in better mechanical and electrical properties.

Razal, Wallace and co-workers reported a simplified wet spinning process for the preparation of PEDOT:PSS fibers with promising electrical properties.^141^ The process uses a spinning formulation consisting of an aqueous mixture of PEDOT:PSS and polyethylene glycol. The PEDOT:PSS spinning formulation was pretreated with polyethylene glycol and used during wet spinning with a suitable coagulation phase. It was shown that PEDOT:PSS fibers with improved conductivity can be produced continuously in one step. A 30-fold improvement in conductivity compared to that of untreated fibers was achieved. In particular, this one-step approach significantly improved the redox properties of the fibers. The addition of poly(ethylene glycol) to the PEDOT:PSS formulation delayed coagulation and the resulting fibers had more time to elongate before solidifying. There was improved molecular alignment of the PEDOT chains along the fiber axis and their reinforcement toward a bipolaron electronic structure. In-situ electrochemical electron spin resonance spectroscopy revealed improved carrier delocalization.

**Figure 8:**
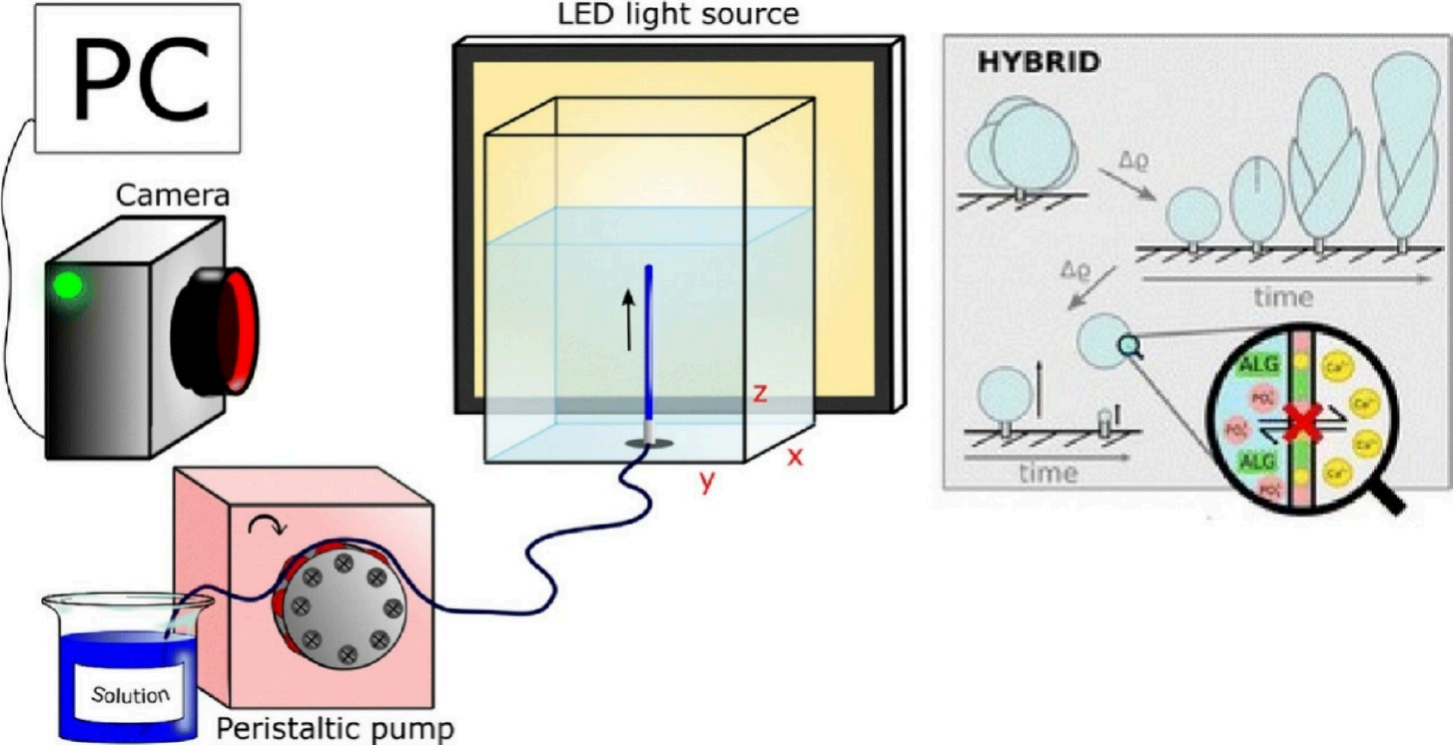
Preparation of tubular budding calcium alginate hydrogels and their inorganic phosphate complexes achieved by the addition of inorganic salts using the flow injection technique, a spinning-type process. Reprinted with permission from ref (142). Copyright 2022 Royal Society of Chemistry.

The spinning process can be used to synthesize hybrid structures of organic and inorganic materials as well as polymeric materials. Tóth and co-workers have produced tubular budding calcium alginate hydrogels and their inorganic phosphate complexes (Figure 8).^142^ The formation of hybrid organized structures is achieved by the addition of inorganic salts using the flow injection technique, which is a spinning-type process. Different flow conditions induce a nonequilibrium system that self-organizes into spatiotemporal structures. The viscoelastic properties of the developed membrane are controlled by the injection rate, and its thickness is controlled by the amount of sodium phosphate in addition to diffusion. The potential difference and gradient are controlled depending on the properties of the resulting structure. The ratio of inorganic to organic content also significantly affects the material properties of the synthesized tubular hydrogel structures. The design and control of new hybrid materials with ionic compartmentalization can also contribute to the synthesis of industrially important materials.

**Figure 9:**
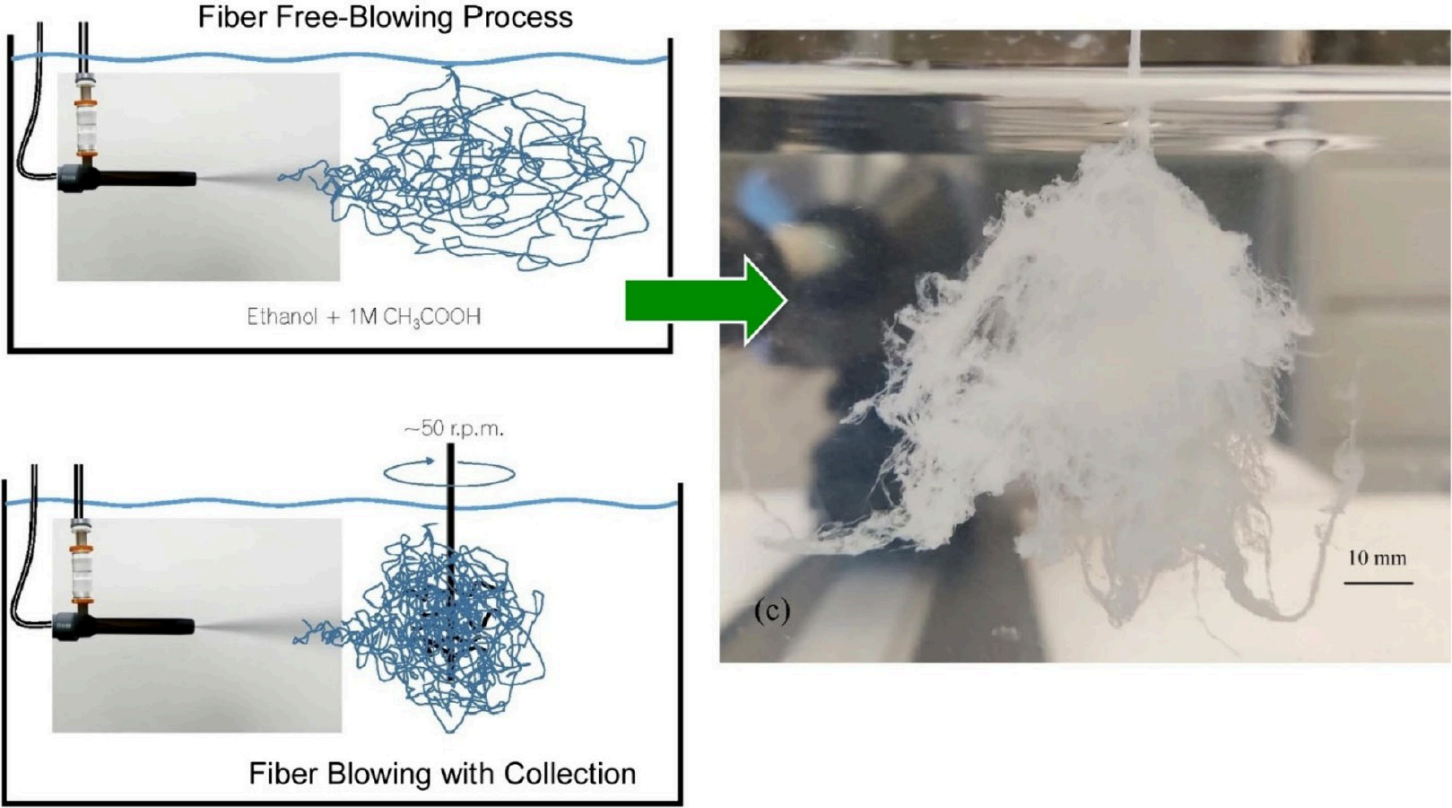
Mass produce entangled fibrous materials consisting of silk fibers from concentrated solutions under hydrodynamic conditions of turbulent flow. Reproduced under terms of the CC-BY license from ref (145), 2022 Springer-Nature.

Structures and organizations of biological materials often serve as models for the design of artificial materials.^143,144^ For example, natural structural materials possess a unique combination of strength and toughness as a result of complex hierarchical assembly across multiple length scales. Gañán-Calvo et al. have developed an assembly process that takes advantage of turbulence (Figure 9).^145^ In this method, the hydrodynamic conditions of turbulent flow can be used to mass-produce entangled fibrous materials consisting of silk fibers from concentrated solutions. A turbulent liquid jet was formed by coflowing a chemically green and simple coagulation liquid (dilute solution of acetic acid in ethanol) with a concentrated aqueous solution of fibroin using a flow blurring nebulizer. In this system, flowing coagulation fluid extracts water from the original protein solution. At the same time, self-assembled proteins are subjected to mechanical actions such as splitting and stretching. The stress distribution characteristic of the turbulent state is reflected in the distribution of the equivalent fiber diameters over approximately 3 orders of magnitude. The pulling action can also be used to assemble fibers into cotton-like objects using a rotating brush. The resulting fibers are subjected to high and relatively uniform stresses along their spans. These stresses can overcome the applied macroscopic pressures that drive the complex flow of the dope and the coflow by several orders of magnitude. The resulting material is 100% biocompatible and, therefore, highly suitable for the development of large scale, low cost, green, and sustainable bioapplications.

**Figure 10:**
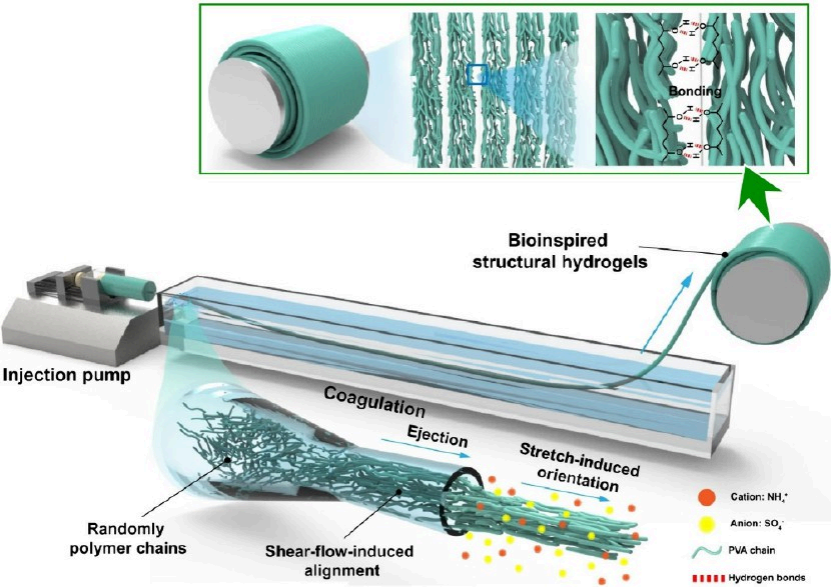
A schematic of the continuous fabrication process for developing hierarchical assemblies of anisotropic structures. Reproduced under terms of the CC-BY license from ref (148), 2024 Springer-Nature.

The structures and organizations of biological materials often serve as models for the design of artificial materials. For example, natural structural materials possess a unique combination of strength and toughness because of the complex hierarchical assembly across multiple length scales.^146,147^ Designing such well-ordered structures in synthetic materials in a universal and scalable manner is not a trivial task. To address such challenges, Lee, Lin and co-workers proposed an approach to design hierarchically structured hydrogels by flow-induced alignment of nanofibrils.^148^Figure 10 shows a schematic of the continuous fabrication process for developing hierarchical assemblies of anisotropic structures. Specifically, a poly(vinyl alcohol) solution is injected into 3 M ammonium sulfate and stretched to form hydrogel fibers. The shear flow controls the orientation of the polymer chains in the spinneret, where a velocity gradient perpendicular to the fluid velocity is distributed. This velocity field gradient generates shear forces that align the polymer chains along the flow direction. Extensional flow in the coagulation bath refers to the velocity gradient along the fluid velocity, which determines the orientation and properties of the polymer. During extensional flow, the polymer chains are stretched and aligned parallel to the flow direction, increasing the degree of molecular orientation in the composite. The resulting stretch-induced alignment is due to the synergistic effect of the densification of the structure and hierarchically aligned fiber structure. The subsequent salting-out process drives the hydration water between the polymer chains, forming a dense network. The resulting microchannel structure provides ultrafast and anisotropic mass transport functions such as water purification. Flow-induced alignment is a strategy for producing bioinspired structural hydrogels with molecular precision. It can also be rapidly produced on a large scale. Synthetic structural materials are expected to have applications in bioengineering, drug delivery, water purification, and soft electronics.

**Figure 11:**
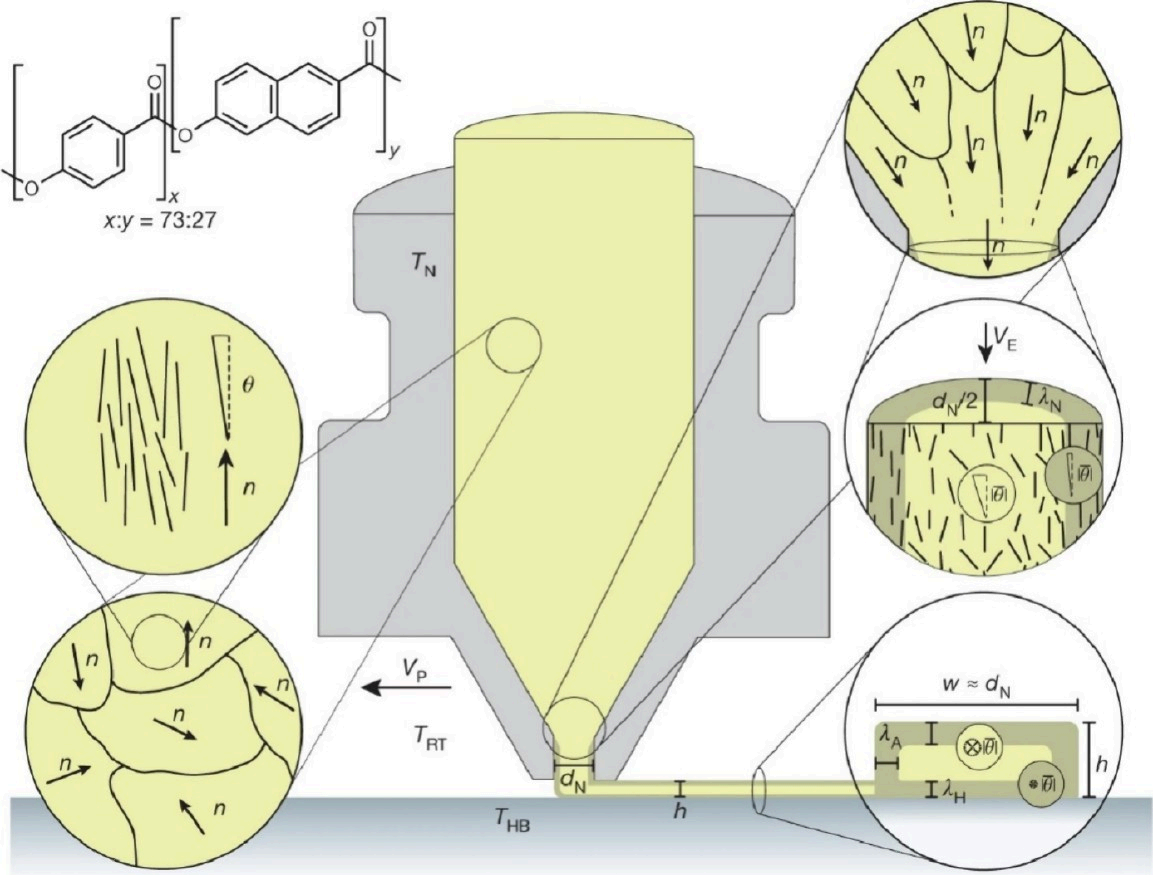
A 3D printing approach by the self-organization of liquid crystalline polymer molecules into highly oriented domains during extrusion of the molten feedstock. Reprinted with permission from ref (152). Copyright 2018 Springer-Nature.

Fiber-reinforced structures are often used where stiff, lightweight materials are required, such as in aircraft, vehicles, and biomedical implants.^149−151^ To organize such materials, they need to be created by directed self-assembly into complex, hierarchically structured shapes with excellent mechanical properties. Masania, Tervoort, Studart and co-workers report a 3D printing approach to produce hierarchical structures, complex shapes and recyclable lightweight structures with unprecedented stiffness and toughness (Figure 11).^152^ The method is characterized by the self-organization of liquid crystalline polymer molecules into highly oriented domains during the extrusion of the molten feedstock. The rigid molecular segments of the material used here, an aromatic thermotropic polyester, self-organize into nematic domains above the melting point of the material. In the original polymer melt, there is no preferred orientation of the individual aligned nematic domains, but rather an overall random orientation distribution. As the polymer melt is extruded from the nozzle of a 3D printer, shear and strain flow fields are created that align the nematic domains in the flow direction. As the molten polymer exits the nozzle, the flow stops and the extruded filament is exposed to ambient temperature. A temperature gradient is then created between the cold surface and hot interior of the filament. As the surface rapidly cools, the nematic order of the flow-aligned configuration solidifies. As the polymer chains inside the filament cool more slowly, they reorient because of thermal motion. The extruded filament has a core–shell structure with a highly oriented skin surrounding a less oriented core. The produced core–shell filament exhibited remarkable mechanical strength and modulus. Combining the top-down freedom of 3D printing with the bottom-up molecular control of polymer orientation provides the freedom to design and realize structures without the constraints typical of the current manufacturing processes. This opens up the possibility of producing structures that meet different performance requirements, while ensuring a sustainable material life cycle.

**Figure 12:**
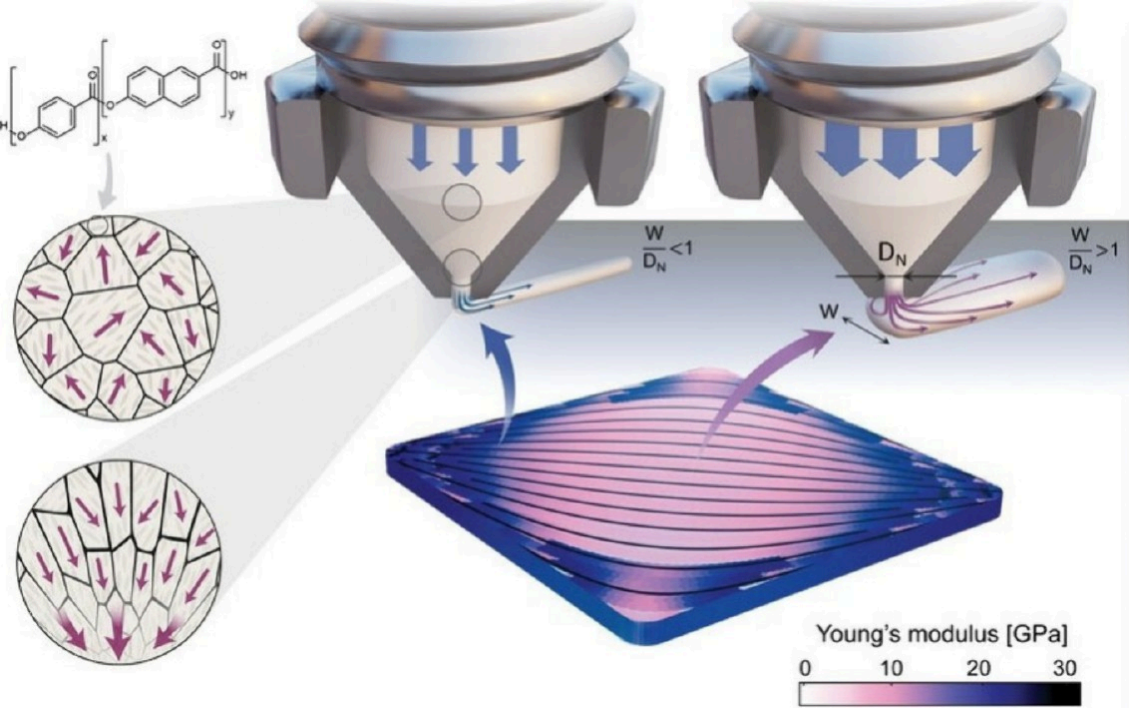
A 3D printing of flow-inspired anisotropic patterns with liquid crystalline polymers, where functional objects with stiffness and curvature gradients can be regulated. Reprinted with permission from ref (153). Copyright 2024 Wiley-VCH.

In a similar approach, Masania and co-workers report that a surprisingly wide range of elastic moduli can be obtained by exploiting the directionality of the nematic flow in the printing process (Figure 12).^153^ Determining the relationship between stiffness, nozzle diameter, and line width identifies a design space where molding and mechanical performance can be combined. By exploiting the synergy between path-planning methods and liquid crystal polymers, functional objects with stiffness and curvature gradients can be 3D printed. Thermotropic liquid crystal polymers, which exhibit self-aligning behavior, are deposited by extrusion 3D printing. Above their melting point, their rigid molecular segments self-organize into nematic domains that are several micrometers in size. Anisotropy is created in the melt by shear and extensional flows that occur during extrusion from the converging nozzle of the 3D printer. When the polymer exits the nozzle and is exposed to ambient temperature, the liquid crystal orientational order within the nematic domains is maintained. This technique has potential applications in lightweight and sustainable structures that incorporate crack mitigation strategies. The wealth of manufacturable patterns expands the design space of possible functional, lightweight objects with stiffness and curvature gradients. This feature may open research avenues to reproduce and study complex natural patterns such as turbulent flow and biological microstructures such as wood and bone.

**Figure 13:**
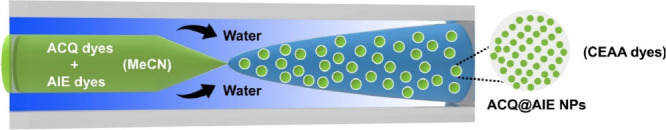
A schematic diagram of the preparation of aggregation-caused quenching dyes using a continuous flow microreactor. Reproduced under terms of the CC-BY license from ref (157), 2022 Springer-Nature.

The flexible fabrication of nanoparticles is also an important challenge in the development of functional materials.^154−156^ The rational fabrication of nanoparticles can also lead to the realization of ideal light-harvesting systems at low cost. Tao and co-workers reported the synthesis of a hybrid dye nanoparticle system based on organic dyes encapsulated in tetraphenylene in a continuous flow microreactor.^157^Figure 13 shows a schematic diagram of the preparation of aggregation-induced emission (AIE) and aggregation-caused quenching dyes (AQC) using a continuous flow microreactor. Composite dye nanoparticles realize the coemission of aggregation-induced emission dyes and aggregation-caused quenching dyes (CEAA). The inner phase fluid in the capillary is an acetonitrile solution of precursors (tetraphenylene and organic dyes) and the outer square fluid channel is filled with antisolvent water. The precursor solution is discharged through the capillary and precipitates in water to form the aggregation-induced quenching dye. Using a continuous flow microreactor, they prepared ACQ@AIE-type nanoparticles (CEAA dyes) that combined the properties of aggregation-caused quenching and aggregation-induced emission. The preparation of CEAA can be controlled by the concentration difference between different dyes, and CEAA realizes the coemission of ACQ dyes and AIE dyes in the same nanoparticles. This nanoparticle system has an ultraefficient light-harvesting ability with a long red-shift distance. The efficient light-harvesting ability is achieved because the organic dyes are uniformly dispersed and immobilized within the AIE solid particles. The necessary conditions for a stable Förster resonance energy transfer (FRET) process are met, an efficient cascade FRET process is achieved, which contributes to the functionality of the nanoparticles. The demonstrated CEAA dyes may contribute to biomimetic artificial light-harvesting.

**Figure 14:**
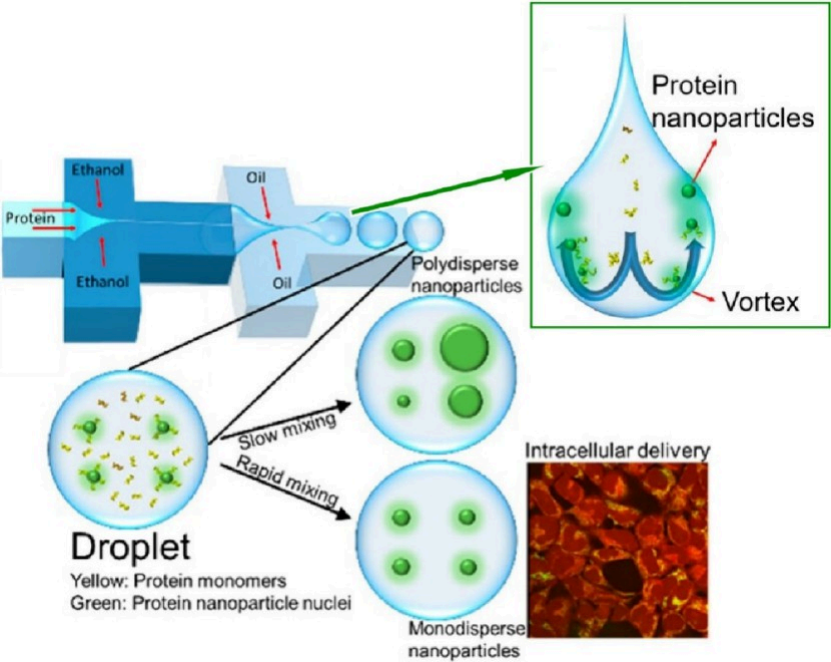
Droplet microfluidics to generate highly monodisperse protein nanoparticles by exploiting the properties of rapid and continuous mixing within microdroplets. Reproduced under terms of the CC-BY license from ref (161), 2023 American Chemical Society.

Nanoparticles are increasingly being used for biological applications such as drug delivery and gene transfer.^158−160^ A variety of biological and bioinspired building blocks, including lipids and synthetic polymers, have been used to generate such particles. Proteins are an attractive class of materials for such applications because of their excellent biocompatibility, low immunogenicity, and self-assembly properties. Wang, Knowles and co-workers used droplet microfluidics to generate highly monodisperse protein nanoparticles by exploiting the properties of rapid and continuous mixing within the microdroplets (Figure 14).^161^ This method exploits naturally occurring vortex flows within microdroplets to prevent nanoparticle aggregation after nucleation and to systematically control particle size and monodispersity. They use a droplet microfluidic device in which oil flows as the outer phase, ethanol as the middle phase, and protein as the inner phase. Ethanol reduces the solubility of protein molecules and acts as an effective desolvation agent, leading to protein nucleation and ultimately the formation of nanoparticles. The oil phase separates the reaction solution into aqueous droplets, and the high level of mixing within the droplets allows for faster molecular interactions within the droplets, which in turn promotes the formation of monodisperse nanoparticles. By exploiting the rapid and continuous mixing of liquids within microdroplets, monodisperse and uniform nanoparticles can be produced in a high-throughput manner. As an example, nanoparticles were found to be highly biocompatible with HEK-293 cells. Confocal microscopy confirmed that the nanoparticles completely penetrated the cells and were taken up by almost all of them. Nanoparticles can be prepared from various proteins such as silk fibroin, bovine serum albumin, and beta-lactoglobulin. In addition, by integrating RNA or drug molecules into the aqueous phase, protein nanoparticles produced by this microfluidic method can be used in biotechnological fields such as intracellular drug delivery and transgenic delivery.

**Figure 15:**
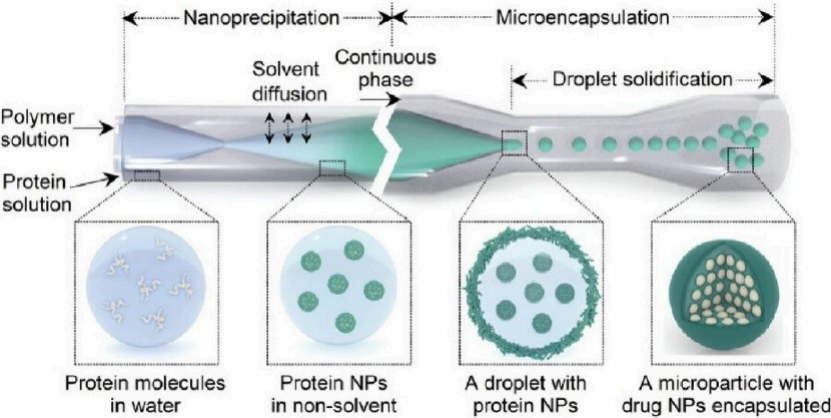
Formation of single microparticles with high drug loading and controlled payload release by controlling the interfacial distribution of the polymer under continuous using a microfluidic platform. Reproduced under terms of the CC-BY license from ref (162), 2023 Wiley-VCH.

Santos, Liu and co-workers developed a single microparticle with high drug loading and controlled payload release by controlling the interfacial distribution of the polymer under continuous flow (Figure 15).^162^ A microfluidic platform offering optimal control of the synthesis process and continuous preparation capability was used to prepare the payload nanoparticles and corresponding microparticles. To overcome poor miscibility with the carrier material, protein molecules were converted into nanoparticles whose surfaces were covered with polymer molecules. In particular, polymers with charged functional groups have been used to promote electrostatic attraction to oppositely charged nanoparticles, thus achieving universal surface decoration of cargo nanoparticles. Considering the effect of electrostatic forces on the surface adsorption, spermine-modified acetylated dextran, a cationic polymer rich in amino groups, is selected as the carrier material. The polymer layer impedes the transfer of cargo nanoparticles from oil to water, achieving excellent encapsulation efficiency (up to 99.9%). The resulting droplets are converted into solidified microparticles after solvent depletion in the oil phase. The microparticles were successfully designed by controlled interfacial self-assembly of polymers to achieve ultrahigh drug loading and zero-order release of the protein payload.

**Figure 16:**
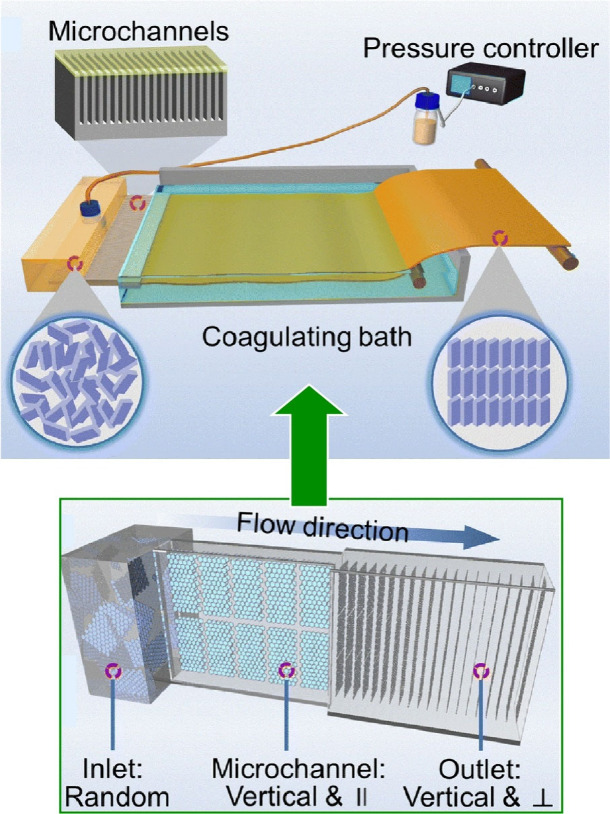
A scalable and efficient microfluidic-based sheet alignment process for assembling 2D nanosheets into large-area films with highly ordered vertical alignment. Adapted with permission from ref (166). Copyright 2023 Royal Society of Chemistry.

2D nanosheets have been assembled into various macrostructures for a wide range of engineering applications.^163−165^ To fully exploit their excellent thermal, mechanical and electrical properties, 2D nanosheets must be aligned into highly ordered structures owing to their strong structural anisotropy. Wang, Xin and co-workers report a scalable and efficient microfluidic-based sheet alignment process for assembling 2D nanosheets into large-area films with a highly ordered vertical alignment (Figure 16).^166^ Using a high aspect ratio microchannel array, the proper vertical alignment of randomly oriented 2D nanosheets is achieved under the severe channel size constraint imposed by high shear stress. When graphene oxide nanosheets undergo a microfluidic-based sheet alignment process, the aqueous graphene oxide dispersion is pumped through the microchannel array device into a coagulation bath. While flowing in the microchannel, the non-Newtonian graphene oxide dispersion forms a large-volume plug flow in which the graphene oxide fluid moves forward, similar to a solid. The low velocity gradient in the plug flow leads to weak shear stress, resulting in the formation of randomly oriented 2D nanosheets. In the microchannel, the randomly oriented graphene oxide nanosheets rearrange into a highly ordered vertical configuration, with the basal plane of the sheets parallel to the microchannel sidewall. The continuous flow process is also capable of mass producing vertically oriented sheet structures. The resulting nanosheets were considered for application as thermally conductive fillers to improve the heat transfer efficiency. The highly aligned, vertically oriented graphene sheets provided better cooling performance than commercial thermal pads and brought a record thermal conductivity to thermal interface materials. This method is expected to be extended to MoS_2_ and boron nitride nanosheets. This will greatly facilitate the development of 2D materials for various applications such as energy storage, desalination, thermal management, and structural materials.

**Figure 17:**
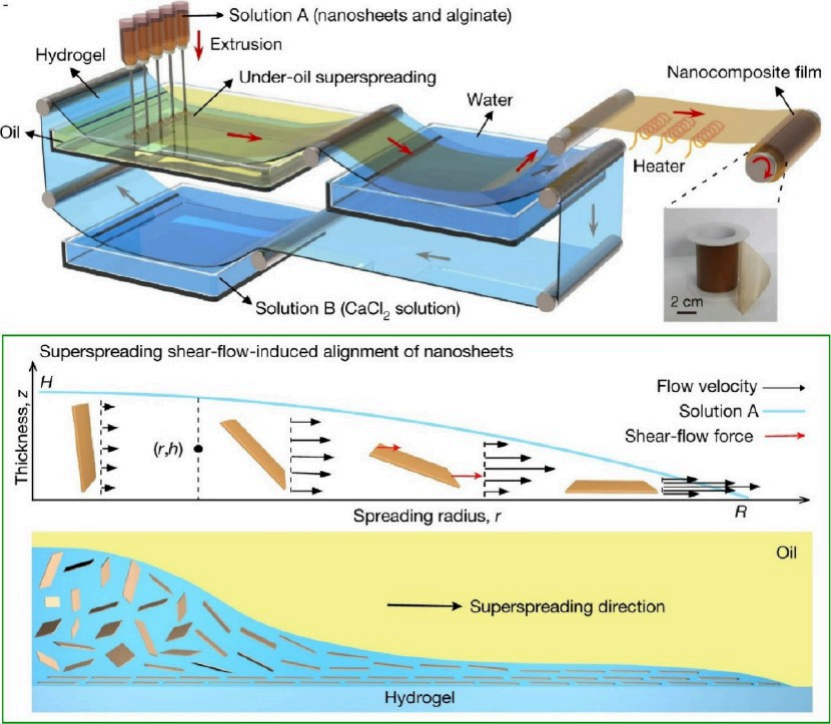
A strategy to fabricate nanocomposites with highly ordered layered structures using shear flow-induced alignment of 2D nanosheets at an immiscible hydrogel/oil interface. Adapted with permission from ref (170). Copyright 2020 Springer-Nature.

Biological materials such as bones, teeth and mollusk shells are known for their exceptional strength, modulus and toughness.^167−169^ Such properties are attributed to the exquisite layered microstructure of 2D nanosheets or nanoplatelets. As a universal, feasible, and scalable method for fabricating ultrastrong layered nanocomposites, Liu and co-workers developed a strategy to fabricate nanocomposites with highly ordered layered structures using shear flow-induced alignment of 2D nanosheets at an immiscible hydrogel/oil interface (Figure 17).^170^ The fluid flow can promote the oriented assembly of nanofillers by controlling the forward or backward motion of the three-phase contact line. In an oil/water/gel system, there is a superspreading phenomenon where droplets spread rapidly and completely on a miscible gel surface. A droplet of a reaction solution containing graphene oxide nanosheets and sodium alginate achieves superspreading within 358 ms on the surface of a fully swollen polyacrylamide hydrogel under silicone oil. They developed a method to extend the superspreading process to a continuous system by simultaneously extruding the reacting solutions using a series of syringes. This method produced large-area nanocomposite films with aligned nanosheets. This is a generalizable and scalable lamination method based on the superdiffusive shear flow-induced alignment of nanosheets at an immiscible hydrogel/oil interface. It can be easily extended to align a variety of two-dimensional nanofillers and is applicable to a wide range of structural composites, potentially leading to the development of high-performance composites. We anticipate that this superdiffusive lamination strategy will be more widely used in the development of advanced layered nanocomposites for practical applications.

In this section, we present examples of the modulation of structural organization by the forced flows designed in various devices and processes. The macroscopic flows and stresses are controlled easily, the use of which allows a straightforward approach to control nanoscale structures. Further explore of the target applications and materials may lead to novel designs of processes using forced flows in future.

## 4 Organization by Flows at Interfaces

In the previous two sections, we outlined examples of material deaminization caused by flows owing to natural phenomena and artificial forced flows. In the following section, we change our perspective and focus on the field (media). In particular, we consider interfaces, which are important domains in the fabrication of many structures. Interfacial science techniques are used to organize materials at interfaces. Examples include self-assembled monolayer (SAM),^171,172^ layer-by-layer (LbL) assembly,^173−175^ and the Langmuir–Blodgett (LB) method.^176−178^ Of these, SAMs are strongly constrained to the substrate, but flow effects are more likely to occur in LbL multilayer film fabrication and film formation on the liquid surface in the LB method. With this in mind, in this section, we discuss several examples of thin film fabrication using LbL assembly and the LB method.

### 4.1 Layer-by-Layer (LbL) Assembly

**Figure 18:**
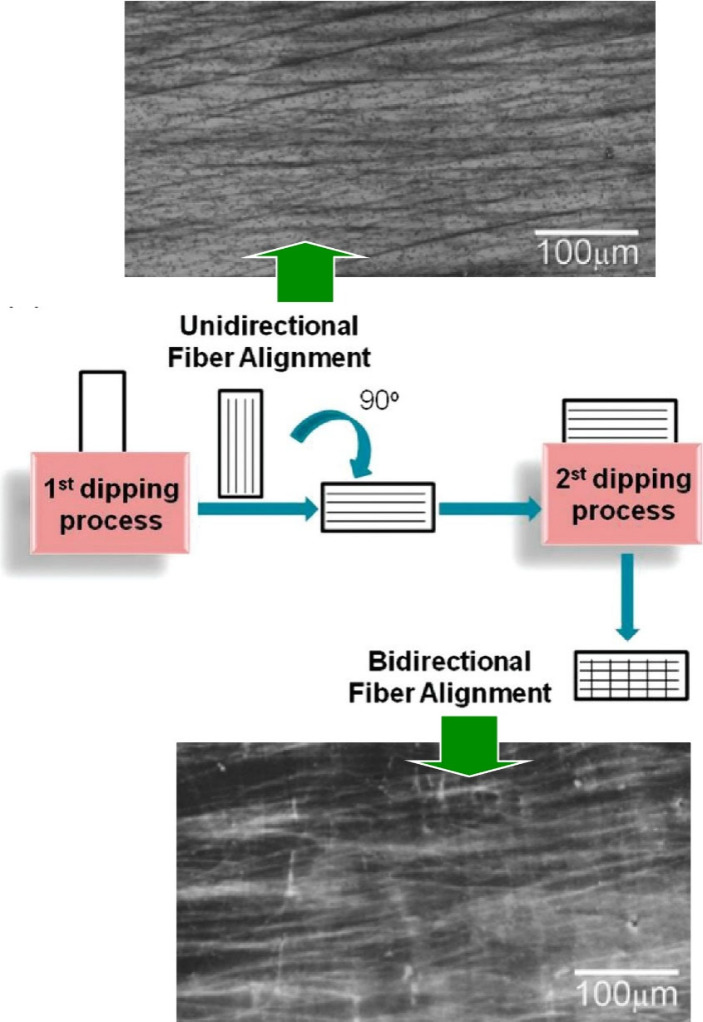
Controlled the orientation of chitosan/silk fibroin layer by layer multilayer thin films layer by layer, where dipping direction can be changed by rotating the substrate during deposition. allowing fiber orientation from one direction to two directions. Adapted with permission from ref (180). Copyright 2010 American Chemical Society.

LbL assembly is a method of building materials in a layer by layer fashion on a substrate based on the interactions between materials. A large number of materials can be used for LbL assembly. This versatile method is also called fuzzy nanoassembly and is a flexible and ambiguous lamination method.^179^ It can also be considered as a thin-film fabrication method that is susceptible to flow and stress. For example, Rubner and co-workers controlled the orientation of chitosan/silk fibroin LbL multilayer thin films (Figure 18).^180^ This LbL assembly process produces fibers that are aligned in the direction of immersion. The direction can be changed by rotating the substrate during the deposition. This allows fiber orientation from one direction to two directions. The detailed fiber deposition and orientation can be controlled by careful choice of solvent and drying conditions. It has been shown that silk fibroin adopts a silk II secondary structure in the laminated LbL films. These anisotropic films can combine the biocompatibility of natural polymer systems with the mechanical strength of silk fibroin. The ability to form and control these fibers makes them attractive candidates for surface functionalization in various biomaterial applications. They may be useful in many biological applications, including suitable scaffolds for directing cell adhesion and spreading. They may also be useful in applications such as biomaterial coatings to improve the biocompatibility of implant devices, to direct cell adhesion and growth, and as biotemplates for oriented nucleation and growth of inorganic crystals such as calcium phosphate.

**Figure 19:**
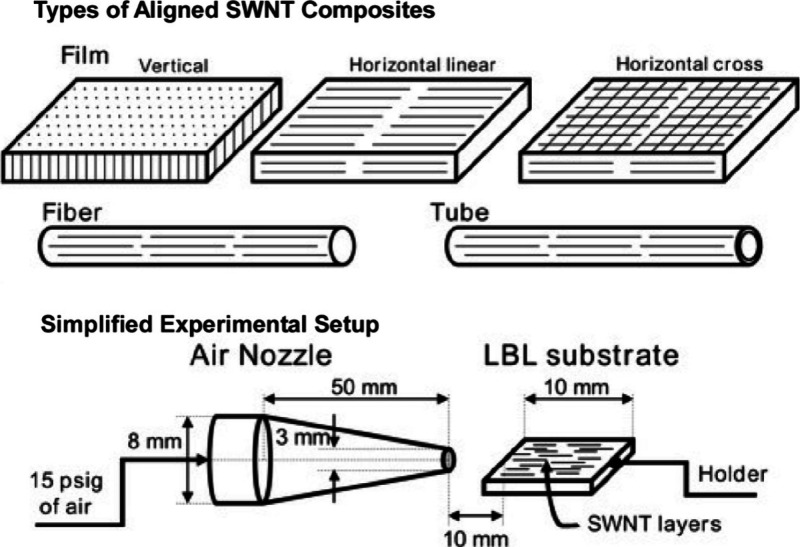
A method to produce aligned SWNT-polymer composites using LbL assembly method, where the alignment of the SWNTs is controlled upon drying steps after washing off excess SWNTs on the poly(vinyl alcohol) surface. Reprinted with permission from ref (184). Copyright 2005 American Chemical Society.

Controlling the orientation of single-walled carbon nanotubes (SWNTs) in polymer composites results in anisotropic shapes and properties.^181−183^ This can dramatically improve the physical properties and performances of the composites. Shim and Kotov developed a method to produce aligned SWNTs and robust SWNT-polymer composites using SWNT combing and LbL assembly fusion methods (Figure 19).^184^ These SWNTs wrapped in poly(styrenesulfonate) are used as LbL assembly components. Poly(vinyl alcohol) serves as the LbL partner, enabling sequential adsorption. On a charged substrate, these two components are assembled by performing a dip treatment on each component, followed by an intermediate washing step to remove excess nonadsorbed components from the surface, and a drying step to stabilize the newly formed molecular layer. The alignment of the SWNTs is controlled during these intermediate drying steps after washing off the excess SWNTs on the poly(vinyl alcohol) surface. When pressurized air is blown into, the SWNTs are stretched by interfacial force. Analysis of the SWNT alignment properties based on the stretching theory shows that the excess drag force of the receding air–water meniscus and the intrinsic surface dewetting rate are essential for SWNT alignment. The resulting aligned SWNTs can offer anisotropic mechanical responses that contributes to the actuation of SWNT-polymer composites and other biomedical and electronic applications. For instance, leveraging the biocompatibility of SWNT composites, they can be exploited to control mammalian cells growing along the direction of SWNT alignment using directional electrical potential in culture.

**Figure 20:**
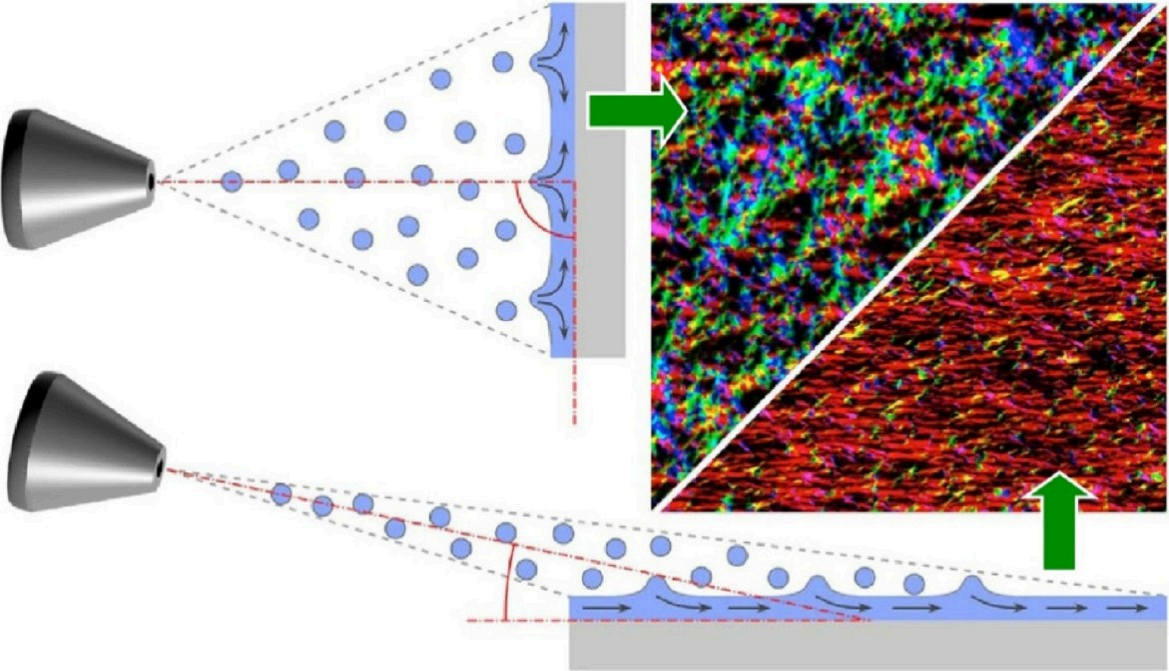
Controlled the orientation of cellulose nanofibrils in LbL assembly films by spray-assisted orientation where cellulose nanofibrils can be easily oriented by oblique incidence spraying to produce films. Reproduced under terms of the CC-BY license from ref (185), 2016 American Chemical Society.

A similar technique was applied to oriented thin films of cellulose nanofibrils. Decher and co-workers controlled the orientation of cellulose nanofibrils in LbL assembly films by spray-assisted orientation (Figure 20).^185^ Oriented LbL monolayer and multilayer films of cellulose nanofibrils were assembled using the oblique incidence spray technique with polycations such as poly(ethylenimine) and chitosan. Spraying at 90° to the receiving surface results in films with a uniform in-plane orientation. Spraying at smaller angles leads to macroscopic directional surface flow of the liquid on the receiving surface, forming films with relatively large in-plane anisotropy. In fact, cellulose nanofibrils can be easily oriented by oblique incidence spraying to produce films that are optically birefringent over large areas. The relative brightness of a birefringent material under crossed polarizers depends on the number of layer pairs in the film. This indicates that all individual cellulose nanofibril layers in a multilayer sample contribute to birefringence and are oriented in the same direction. Highly anisotropic materials can also be obtained by combining the spray orientation with layer-by-layer assembly. In addition, LbL assembly allows the use of different compounds in different layers, enabling the preparation of multimaterials with multiple engineering properties, and oblique incidence spraying allows the easy selection of the orientation direction of each layer, enabling the preparation of materials with complex anisotropy.

**Figure 21:**
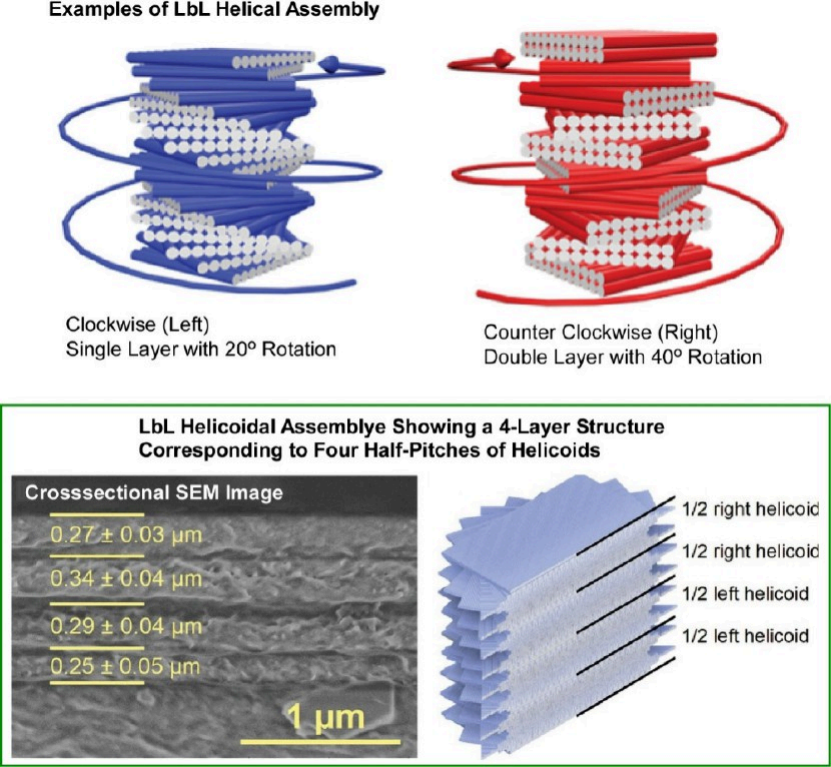
Composite LbL films of cellulose nanofibrils and polyvinylamine with helical alignment assembled by a directed assembly approach with successfully constructing left-handed and right-handed helices with different pitches and rotations with uniform thickness. Reproduced under terms of the CC-BY license from ref (186), 2024 Wiley-VCH.

Attempts have been made to control not only the macroscopic orientation of nanofibrils and nanorods, but also the submicron and chiral structures of their components. Decher, Houérou, Felix and co-workers combined spray-assisted alignment of cellulose nanofibers with LbL assembly.^186^ They proposed an additive manufacturing process that rationally selects the alignment direction of each cellulose layer to realize a thin film with helically aligned nanofibers (Figure 21). Composite LbL films of cellulose nanofibrils and polyvinylamine with helical alignment are assembled using a directed assembly approach. They succeeded in constructing left- and right-handed helices with different pitches and rotations with uniform thickness. The handedness and pitch of chiral structures can be easily tuned by deliberately selecting simple parameters such as the number of successive cellulose layers sprayed in the same direction and the rotation angle between successive layer stacks. This experiment is unique in that it offers the possibility of fabricating complex nanocomposite structures with different nanoscale controlled substructures from different nonisometric objects. This study lays the foundation for the fabrication of nanocomposite films with well-controlled internal structures of the reinforcing fiber phase. Thin films with enhanced mechanical and optical functionalities can be fabricated. Large-area devices can also be fabricated by programming spray sequences using a robotic arm. Such multifunctional biobased composites can be competitive in future applications such as high-performance composites, protective coatings, optical filters, and flexible displays. They can address the environmental and societal needs for sustainable, nonpetroleum-based, high-performance products.

**Figure 22:**
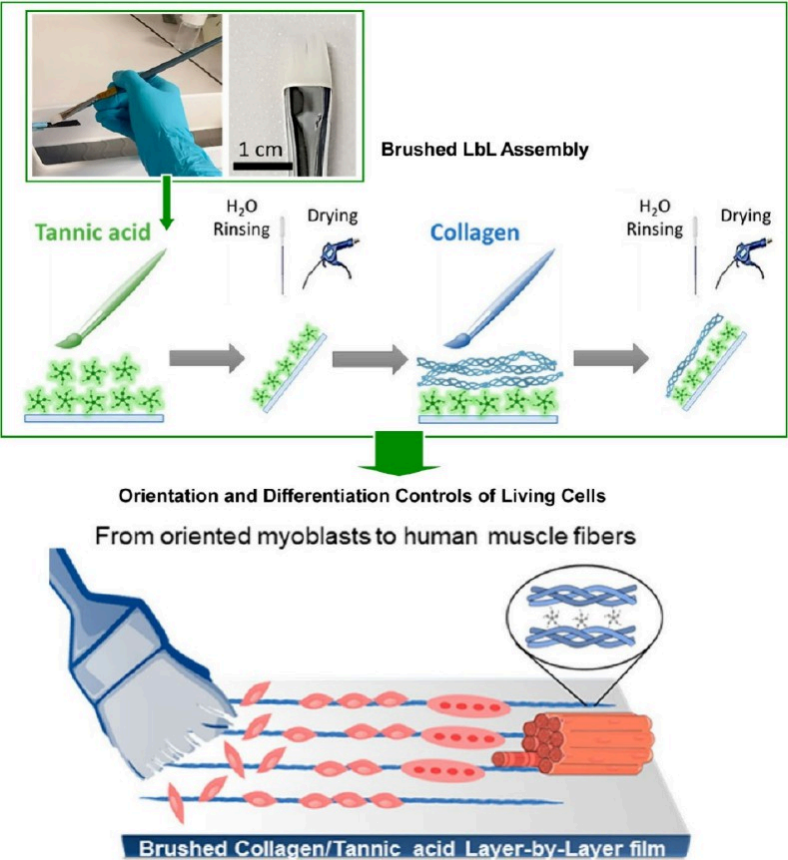
A simple brushing method to prepare hydrogen-bonded tannic acid/collagen LbL nanofilms. Adapted with permission from ref (187). Copyright 2022 American Chemical Society.

In addition to spraying, a simple technique called brushing can be used to control the structure of LbL films. Boulmedais and co-workers used a simple brushing method to prepare hydrogen-bonded tannic acid/collagen LbL nanofilms (Figure 22).^187^ Compared to LbL films obtained by normal dipping, brushed tannic acid/collagen LbL nanofilms have oriented collagen fibers with a diameter of 60 nm along the brushing direction. In addition, oriented tannic acid/collagen LbL nanofilms constructed at acidic pH can release tannic acid into the solution with little loss of thickness upon contact with physiological media. To investigate their functionality, human myoblasts were seeded on the collagen-terminated oriented tannic acid/collagen LbL nanofilms. After 12 days of culture in differentiation medium without other additives, human myoblasts aligned themselves on the brushed tannic acid/collagen LbL nanofilms and differentiated into long, aligned myotubes (from hundreds of μm to 1.7 mm in length). This phenomenon is due to two distinct properties: collagen orientation, which aligns myoblasts and promotes close contact, and tannic acid release, which promotes differentiation. It exploits topographical cues and strong connections between cells and collagen, mimicking the complexity of in vivo conditions. In particular, brushed LbL films are a powerful and simple way to create surfaces with aligned topography using high aspect ratio polymers such as collagen. It holds promise for anisotropic tissue regeneration, the treatment of injury and disease, and the design of model tissues for pharmacological studies.

### 4.2 Langmuir–Blodgett (LB) Method

The Langmuir–Blodgett (LB) method is a thin film fabrication technique in which a monolayer is spread across a liquid interface, such as an air–water interface, compressed and transferred layer by layer to a solid substrate. In fact, interfaces such as the air–water interface are excellent sites for transmitting macroscopic mechanical stimuli to molecular structures and functions.^188−190^ This interface has a macroscopic spread in the lateral direction, and macroscopic mechanical changes can be induced by compression of the monolayer or flow of the canal phase. The interface is at the molecular level in the thickness direction of the film and changes in molecular association, orientation and conformation can occur within the nanometer scale. Macroscopic mechanical stimuli and molecular structural changes can coexist in an environment such as the air–water interface. In fact, at the air–water interface, molecular machines can be even operated by macroscopic actions using human hands. It has been shown that compression and expansion of the monolayer in the LB method can induce molecular mutations through subtle forces. The air–water interface is an attractive site in which the structure can be controlled by flow. The LB method makes it possible to control the structure of the monolayer in response to the flow at the air–water interface, and to control the structure by the flow when transferring the monolayer from the air–water interface to a solid substrate.

**Figure 23:**
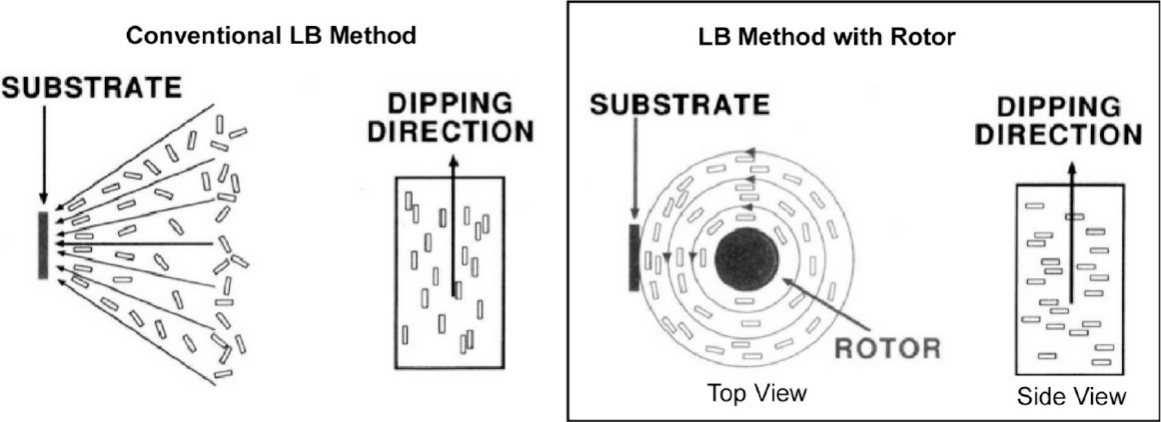
Shearing of the Langmuir film by a rotating disk for the orientation of surfactant molecules at the air–water interface: conventional LB method (left) and LB method with rotor (right). Reprinted with permission from ref (191). Copyright 1995 American Chemical Society.

Attempts have been made to create a flow at the gas–water interface using a rotating disk. As reported by Mingotaud et al., shearing of the Langmuir film by a rotating disk induces the orientation of surfactant molecules at the air–water interface (Figure 23).^191^ Such a monolayer with local anisotropy can be transferred to a solid substrate using the LB technique to obtain a multilayer film with molecular orientation. This orientation process depends mainly on the rotation speed and the distance between the substrate and disk. In the absence of shear, the flow induced by the transfer of the monolayer from the interface to the substrate preferentially orients elongated molecules. The compounds are placed close to the substrate with their long axes approximately parallel. The long axes of the molecules are parallel to the direction of immersion. On the other hand, when the rotating disk is activated, the shear forces cause the molecules to orient themselves at the air–water interface. The major axes of the molecules are parallel to the flow. If the transfer flow is negligible compared to the shear flow, the orientation at the air–water interface is maintained during deposition. The major molecular axes are perpendicular to the direction of immersion. When the two orientation processes compete, not all anisotropy is lost; their influence can be modulated. The advantage of this method is that the dominant molecular orientation in the LB film can be shifted in different directions by simply changing the rotation speed.

**Figure 24:**
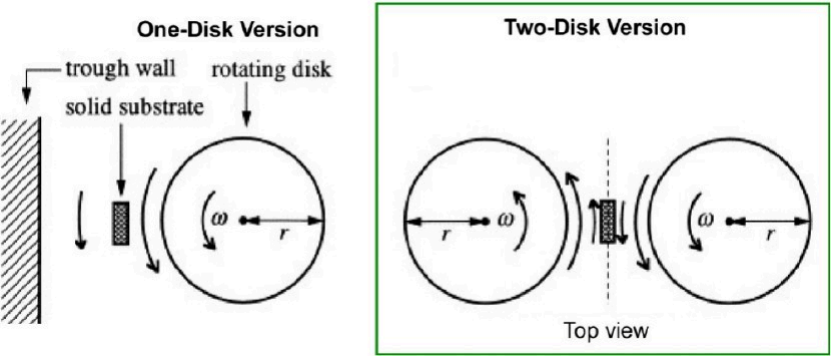
Sheering LB method with a single disk system and a double disk system. Adapted with permission from ref (192). Copyright 1998 Elsevier.

The rotating disk technique can be extended to include the use of multiple rotating disks. Ikegami et al. compared two versions of this method: a single disk system and a double disk system (Figure 24).^192^ In both cases, if the monolayer contains mesoscopic domains with elongated shapes, shear causes them to rotate around their center of gravity. If the shear rate is sufficiently high, the domains are oriented primarily along the flow lines. The one-disk version consists of a simpler mechanical system and is highly effective for macroscopically homogeneous monolayers with sufficient viscoelastic properties. The rotating disk in the one-disk technique generates a concentric monolayer flow at the air–water interface. The rotation rate of the monolayer decreases with increasing distance from the disk. In comparison, for some heterogeneous monolayers, the two-disk version may be more effective at inducing in-plane organization. The rotating disks can provide a simple shear field in the central region between the disks. In other words, the two-disk version requires a more complex mechanical system. However, in all cases, the two-disk version appears to be more suitable for orientation control than the single-disk version.

**Figure 25:**
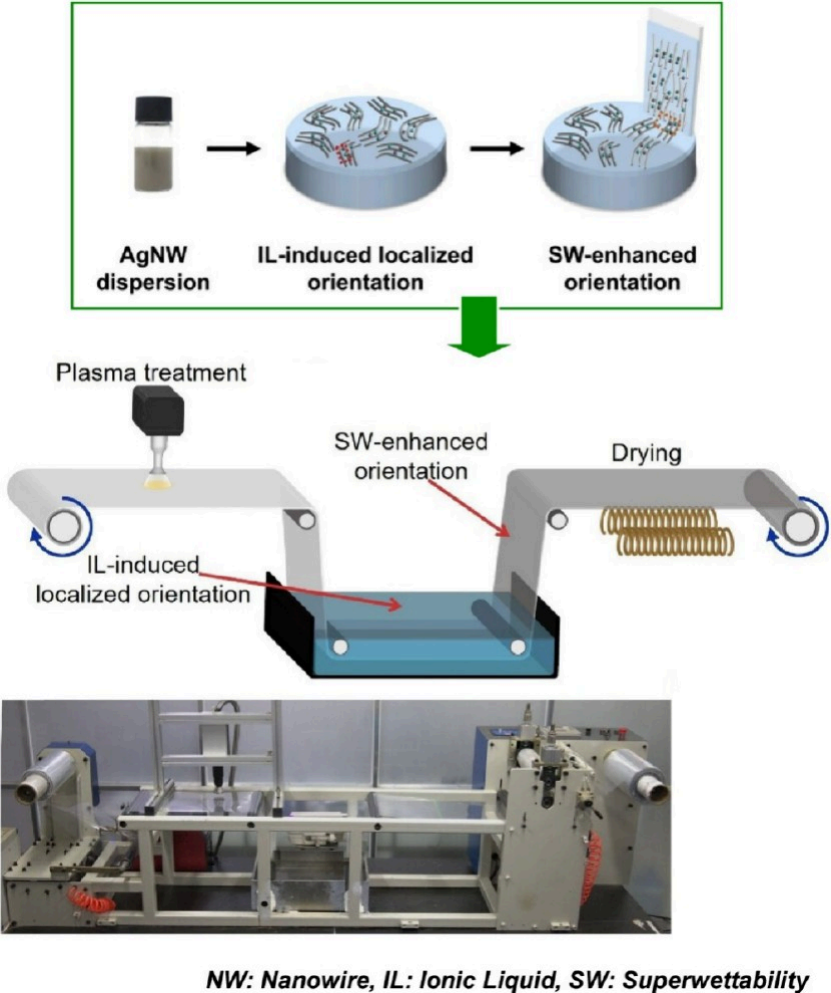
Ionic liquid-induced local alignment at the air–water interface and superwetting enhanced alignment induced by subsequent spontaneous transfer. Adapted with permission from ref (193). Copyright 2024 American Chemical Society.

Aligned and cross-aligned silver nanowires are useful for fabricating flexible transparent electrodes for flexible optoelectronic devices. However, the large-scale production of aligned silver nanowires is not always straightforward. To address this issue, Liu, Diao and co-workers exploited the synergistic effect of ionic liquid-induced local alignment at the air–water interface and superwetting-enhanced alignment induced by subsequent spontaneous transfer (Figure 25).^193^ This provides a facile and scalable strategy to fabricate large-area aligned silver nanowires through the synergistic effect of ionic liquid-induced local alignment at the air–water interface and superwetting-enhanced alignment during superwetting-induced spontaneous transfer onto various flexible substrates. Owing to the formation of hydrogen bonds between the ionic liquid and the silver nanowires and the hydrophobic interactions between the cations in the ionic liquid, the silver nanowires self-assembled at the air–water interface. As a result, they form a locally aligned continuous film. The self-assembled silver nanowire film at the interface climbs onto the prewetted substrate with an enhanced orientation driven by the spontaneous flow resulting from the surface tension difference. The result is an aligned silver nanowire film. By performing the transfer process twice in orthogonal directions, a flexible transparent electrode based on the cross-aligned silver nanowire networks can be easily fabricated. The cross-aligned silver nanowire flexible transparent electrode is known to be applicable in flexible electronic devices such as transparent heaters, electroluminescent devices, and touch panels, and exhibits high photoelectric performance. In addition, it is expected to open new avenues for the large-scale production of other nanowire-based applications.

**Figure 26:**
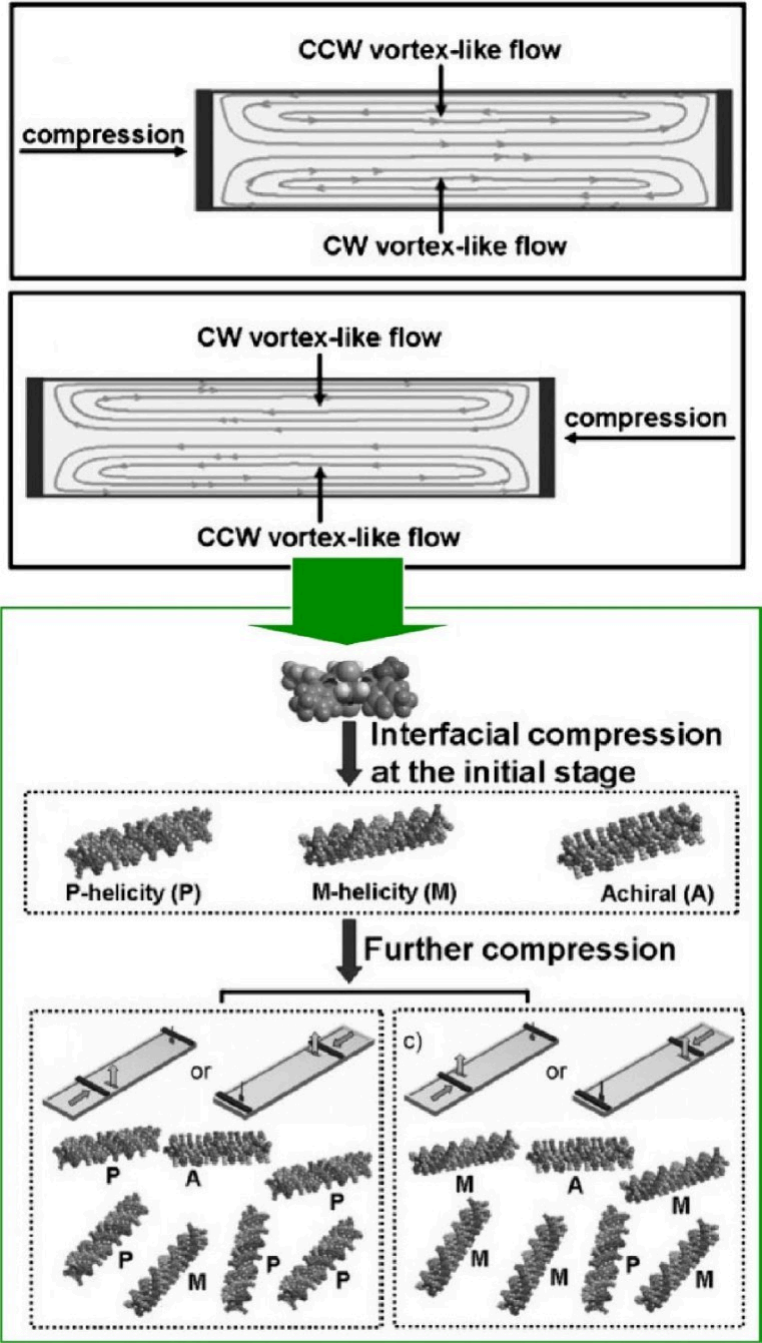
Macroscopic chirality of interracially organized molecular assemblies of achiral porphyrins through the direction of the vortex-like flow generated by compression using the LB technique. Adapted with permission from ref (194). Copyright 2011 Wiley-VCH.

The chirality of some molecular assemblies composed of completely achiral components can be selected by external forces such as vortex motion. The helical direction of the assemblies can also be determined by the left–right hydrodynamic forces. Liu and co-workers showed that the macroscopic chirality of interracially organized molecular assemblies of achiral porphyrins can be selected by the direction of the vortex-like flow generated by compression using the LB technique (Figure 26).^194^ The macroscopic chirality of the interfacial assemblies is selected based on the direction of the vortex-like flow generated by monolayer compression. In the case of one-sided compression, it was found that depending on the geometry, the assemblies deposited from the mirror region of the LB trough exhibited mirror macroscopic chirality. In the case of a one-sided compression geometry, where the Langmuir barrier is compressed from the left side, clockwise and counterclockwise vortex flows are generated at the front and back of the Langmuir trough, respectively. The direction of the vortex flow generated by this compression was found to determine the macroscopic chirality of the formed assemblies. Furthermore, using the standard sample preparation method with double-sided compression geometry, we found that the samples formed around the left and right Langmuir barriers exhibited opposite macroscopic chirality. When the Langmuir barrier was compressed from the right side, counterclockwise and clockwise vortex-like flows were generated at the front and back of the Langmuir groove, respectively, which controlled the macroscopic chirality. These results indicate that this unique LB technique can achieve mechanically controlled supramolecular chirality induction in interfacial systems. This may provide new opportunities for conventional LB techniques to control the macroscopic chirality of supramolecular systems comprising fully achiral units.

**Figure 27:**
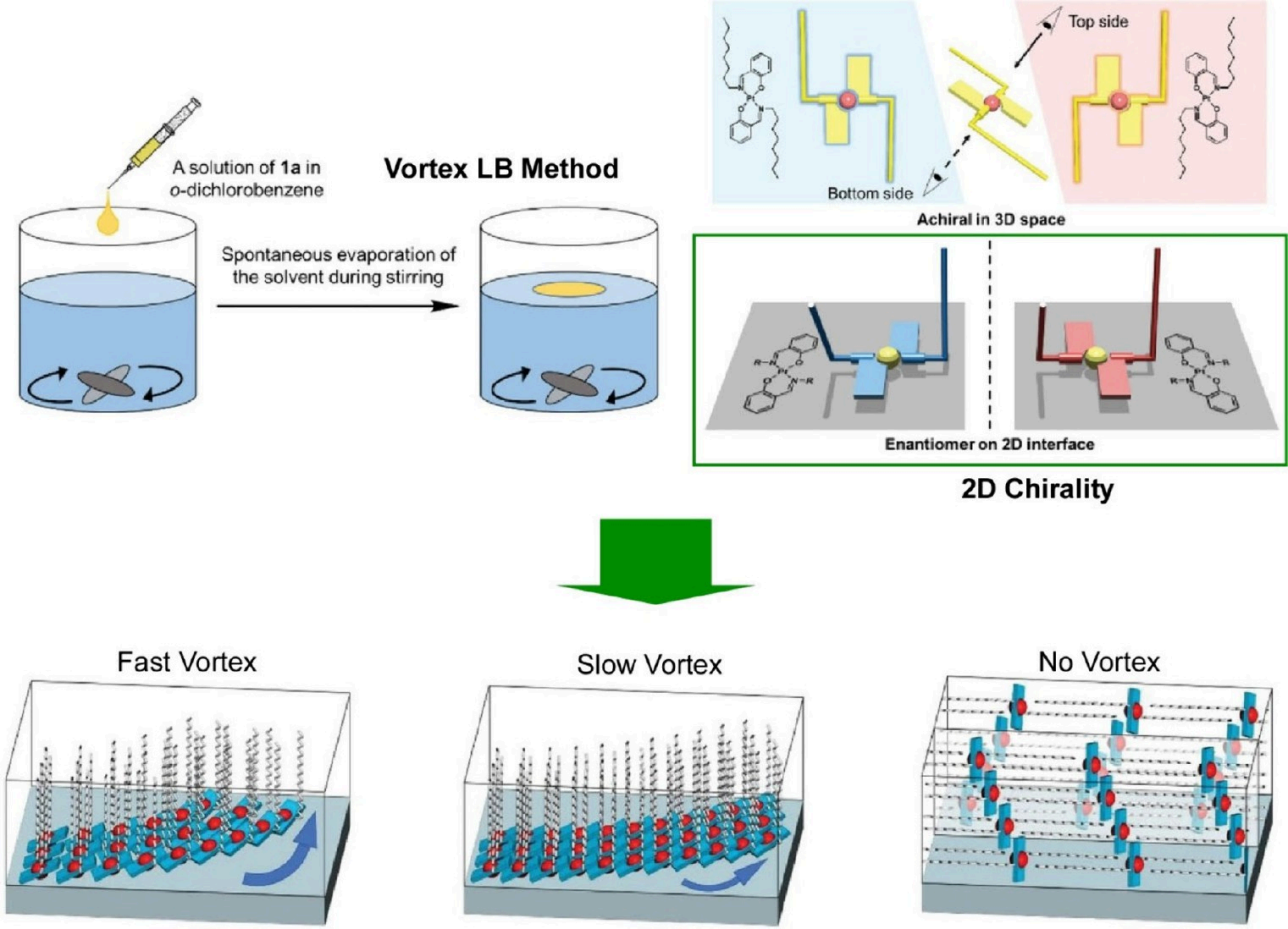
Precise control of the circularly polarized luminescence of aggregates consisting of achiral trans-bis(salicylaldiminato)Pt(II) complexes under vortex flow conditions at the air–water interface. Adapted with permission from ref (195). Copyright 2022 Wiley-VCH.

Circularly polarized luminescence is controlled by properties such as chirality and size. Although attempts have been made to control supramolecular chirality using vortex motion as a mechanical stimulus, precise and reproducible control of circularly polarized luminescence is not always easy. Mori, Muller, Naota and co-workers reported the precise control of the circularly polarized luminescence of aggregates consisting of achiral trans-bis(salicylaldiminato)Pt(II) complexes under vortex flow conditions at the air–water interface (Figure 27).^195^ In the absence of vortex flow, microcrystalline/amorphous solids are formed by thermodynamically controlled 3D aggregation based on a stacked lamellar arrangement of cruciform molecular units. The weak intermolecular interactions and perfect stacking of small aggregates induce the dispersion of light energy in the excited states, leading to nearly nonluminescent properties of the aggregates. The supramolecular chirality of aggregates consisting of Pt(II) complexes is induced and controlled by vortex flow at the air–water interface, whereas the complex spontaneously forms an achiral amorphous solid with nonchiroptical properties under nonvortex flow conditions. The direction and magnitude of the circularly polarized emission of Pt(II) complex aggregates can be precisely tuned depending on the vortex conditions, such as the rotation direction and flow rate. When the vortex flow rate is increased, vortex flow induced emission enhancement is also observed. Under suitable vortex flow conditions, they form kinetically controlled 2D aggregates with a chiral U-shaped conformation at the air–water interface, where the hydrophilic coordination surfaces are attached to the water surface and the hydrophobic *N*-alkyl chains are oriented perpendicular to the water surface by van der Waals forces. It is generated by a unidirectional twist in the stacking of the coordination faces, and the degree of twist is controlled by the vortex flow rate. The Pt coordination faces remain in contact with each other through successive shallow stacking interactions during the chiral transition from a helical twist at the organic-water interface, inducing the enhancement of the circularly polarized and circularly polarized emission properties. The perfect and infinite stacking of the trans-bis(salicylaldiminato)Pt(II) coordination plane causes dispersion of the excited state light energy, and the vortex-driven emission enhancement is attributed to the change in the stacking situation of the coordination plane with the vortex flow velocity. Faster vortex flow leads to the formation of a more helical 2D molecular arrangement with more slippage.

**Figure 28:**
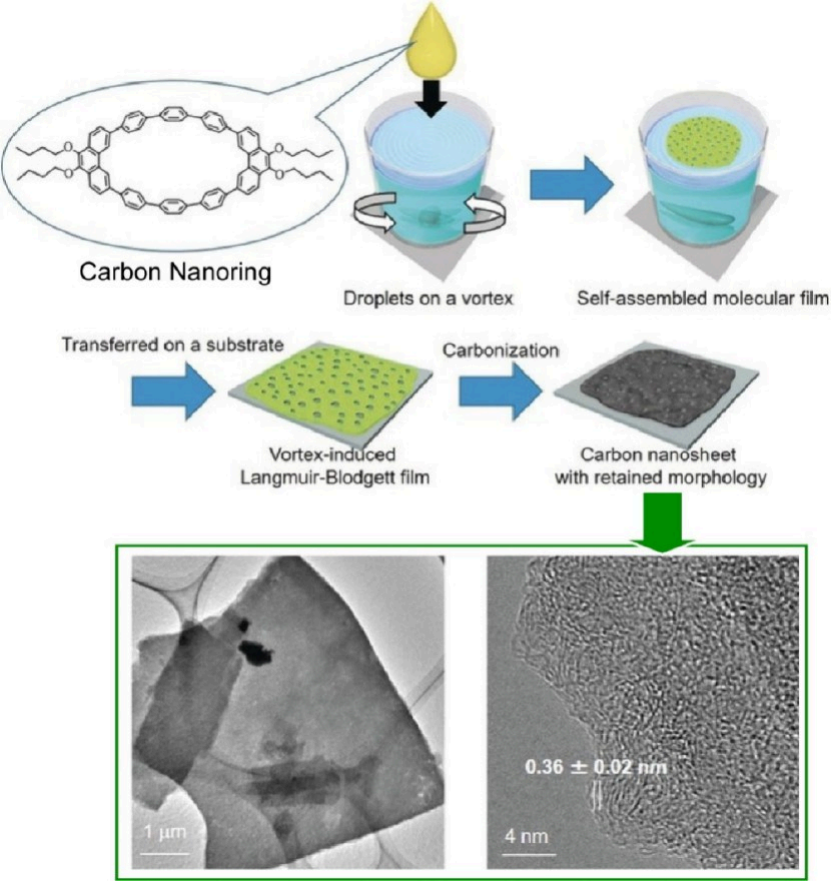
A bottom-up method to synthesize 2D carbon nanosheets using anisotropic carbon nanoring molecules by creating a vortex flow on the water surface. Adapted with permission from ref (199). Copyright 2018 Wiley-VCH.

2D carbon nanomaterials with modulated physical and chemical properties are expected to be used in high-performance energy storage devices and catalysts.^196−198^ However, large-scale fabrication of 2D carbon nanostructures is not always feasible. Mori et al. reported a bottom-up method to synthesize 2D carbon nanosheets using anisotropic carbon nanoring molecules by creating a vortex flow on the water surface (Figure 28).^199^ The carbon nanoring molecules self-assembled to form molecular nanosheets at the dynamic air–water interface with vortex motion. The molecular nanosheets were then carbonized into carbon nanosheets under inert gas flow. The morphology of the nanosheets was preserved after carbonization. This method can be a general strategy for the bottom-up fabrication of large-area carbon nanosheets that do not require specific carbon templates or precursors with specific chemical reactivities. Furthermore, by adding pyridine as a nitrogen dopant during the self-assembly step, nitrogen-doped carbon nanosheets containing mainly pyridinic nitrogen species were obtained. The amount of pyridinic nitrogen species doped into the 2D nanosheets was unexpectedly large, which may enable the use of N-doped carbon nanosheets as highly efficient catalysts for the oxygen reduction reaction in high-performance fuel cells.

**Figure 29:**
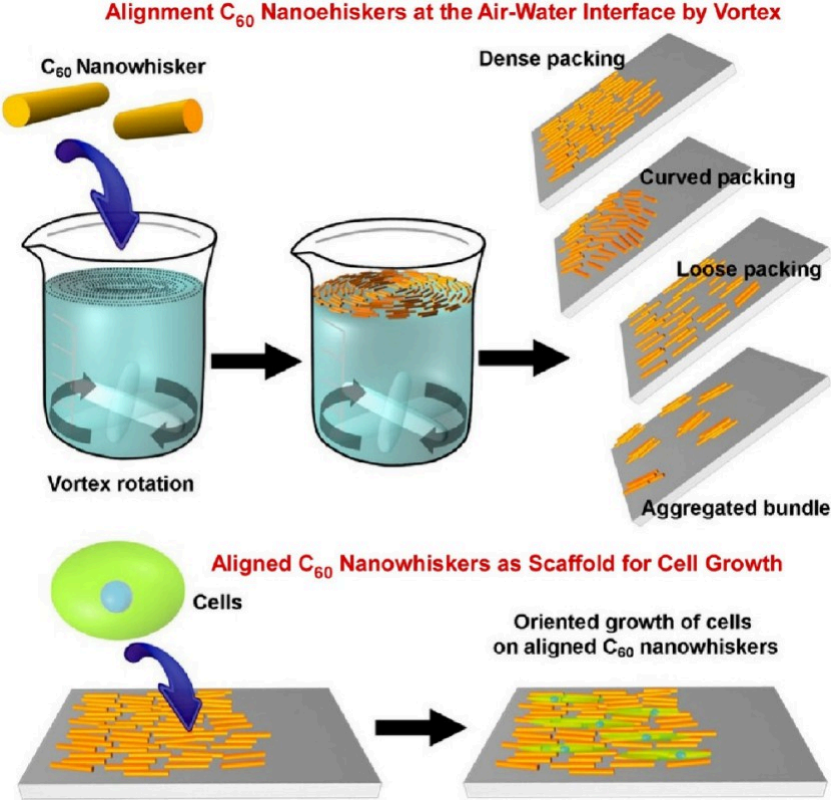
Fabrication of aligned fullerene C_60_ nanowhisker thin films at the air–water interface using vortex LB and usage of them as a scaffold for cell culture. Reprinted with permission from ref (200). Copyright 2015 American Chemical Society.

A molecular assembly method similar to the LB method that uses vortex flows at the gas–liquid interface is called the vortex LB method. Vortex LB can also be used to form aligned nanomaterial structures. Krishnan et al. reported a method and application for the rapid fabrication of aligned fullerene C_60_ nanowhisker thin films at the air–water interface using vortex LB (Figure 29).^200^ This method is based on the vortex motion of the subphase, and the floating fullerene C_60_ nanowhiskers can be aligned on the water surface according to the direction of the rotating flow. Fullerene C_60_ nanowhiskers aligned on a glass substrate were used as scaffolds for cell culture. The array shape of the fullerene C_60_ nanowhiskers can be controlled by the lifting position from the surface. Lifting in a region far from the center of rotation resulted in highly aligned fullerene C_60_ nanowhiskers parallel to the substrate. On the other hand, lifting in a position close to the center led to a curved array. Human osteoblastic MG63 cells, which form bone, adhere well to fullerene C_60_ nanowhiskers. Growth was observed along the axis of the aligned fullerene C_60_ nanowhiskers. Cell proliferation studies showed the low toxicity of C_60_ nanowhiskers, indicating their potential use in biomedical applications. The packing shape and density of fullerene C_60_ nanowhisker arrays can be easily controlled by varying the amount of fullerene C_60_ nanowhiskers, stirring speed, lifting position, and surface hydrophobicity of the substrate. The alignment process can be a powerful method for forming other types of nanostructures or microstructures into highly organized and well-defined 2D architectures.

**Figure 30:**
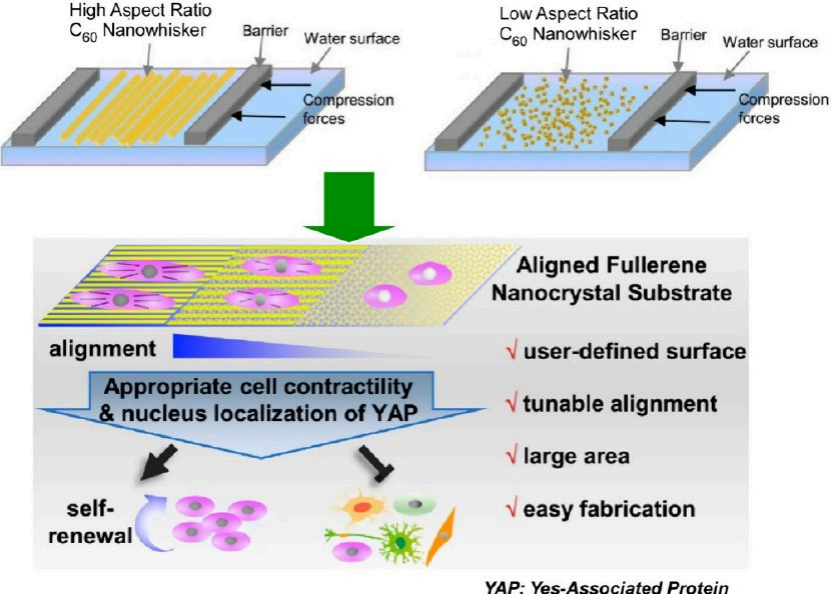
Precise control of hMSC adhesion and differentiation using highly aligned fullerene nanowhisker nanopatterned scaffolds on a solid substrate using the LB technique. Reproduced under terms of the CC-BY license from ref (204), 2020 American Chemical Society.

The development of human mesenchymal stem cell (hMSC)-based therapies has been hampered by the limited availability of adult stem cells.^201−203^ Long-term maintenance of pluripotency and stem-like phenotype during large-scale in vitro expansion processes remains a challenge. To address this challenge, Song et al. reported a prime example of the precise control of hMSC adhesion and function using highly aligned fullerene nanowhisker nanopatterned scaffolds (Figure 30).^204^ The alignment of the fullerene C_60_ nanowhiskers was controlled by a layer-by-layer flow process on a solid substrate using the LB technique. By varying the content of fullerene C_60_ nanowhiskers with different aspect ratios, a large-area nanoarchitectural surface with a continuously adjustable component arrangement was created. The resulting substrate was used for in vitro expansion of hMSCs. The tunable topographical properties of fullerene C_60_ nanowhisker nanopatterns influenced cell spreading, orientation, focal adhesion, and ultimately hMSC self-renewal. Cells cultured on highly aligned fullerene nanowhisker nanopatterned surfaces exhibited long-term maintenance of pluripotency and enhanced regenerative capacity via proper cell contractility and nuclear localization of Yes-associated proteins. This LB approach is a simple technique that can be easily operated manually and therefore can be readily applied in biomedical laboratories for the fabrication of centimeter-sized nanopatterned substrates. This is a promising method for the large-scale expansion of hMSCs in clinical settings. This study provides useful guidance for improving the potential of hMSC technology for regenerative therapy. Such nanotopographical features for stem cell proliferation with enhanced regenerative capacity could be incorporated into future tissue engineering scaffolds.

In these two sections, we present examples of flow-based material organization in an interfacial environment. In particular, we illustrated systems using typical interface processes, namely, LbL assembly and the LB method. These methods are well established, and although technically simple, they are flexible and allow the incorporation of flow elements in different ways. In LbL assembly, highly organized structures can be obtained by combining simple actions such as spraying and brushing. As the LB method is a liquid phase fabrication method, elements such as flow and vortex flow can be easily reflected in the structural organization. In these methods, the macroscopic mechanical behavior of the flow and organization of molecules and materials at the interface are rationally coupled. There are advantages to integrating these processes, such as the ability to manually control sophisticated structures, including the chirality of the organization.

### 4.3 Case Study: Organic Semiconductor Films with Interfacial Process

There are various methods of organizing materials through interface processes other than LbL assembly and the LB method.^205−208^ It is impossible to list all of them, but we can present some examples to highlight their possibilities. In this section, we briefly illustrate the interfacial processes of organic semiconductor thin films, which are attracting considerable attention in terms of their applications. For the 2D crystallization of low-molecular-weight organic semiconductor molecules, sophisticated methods such as continuous edge-casting have been successfully utilized.^210,211^ However, the formation of organized thin films from polymeric organic semiconductors is still under development, and flow-assisted nanoarchitectonics would have meaningful contributions.

**Figure 31:**
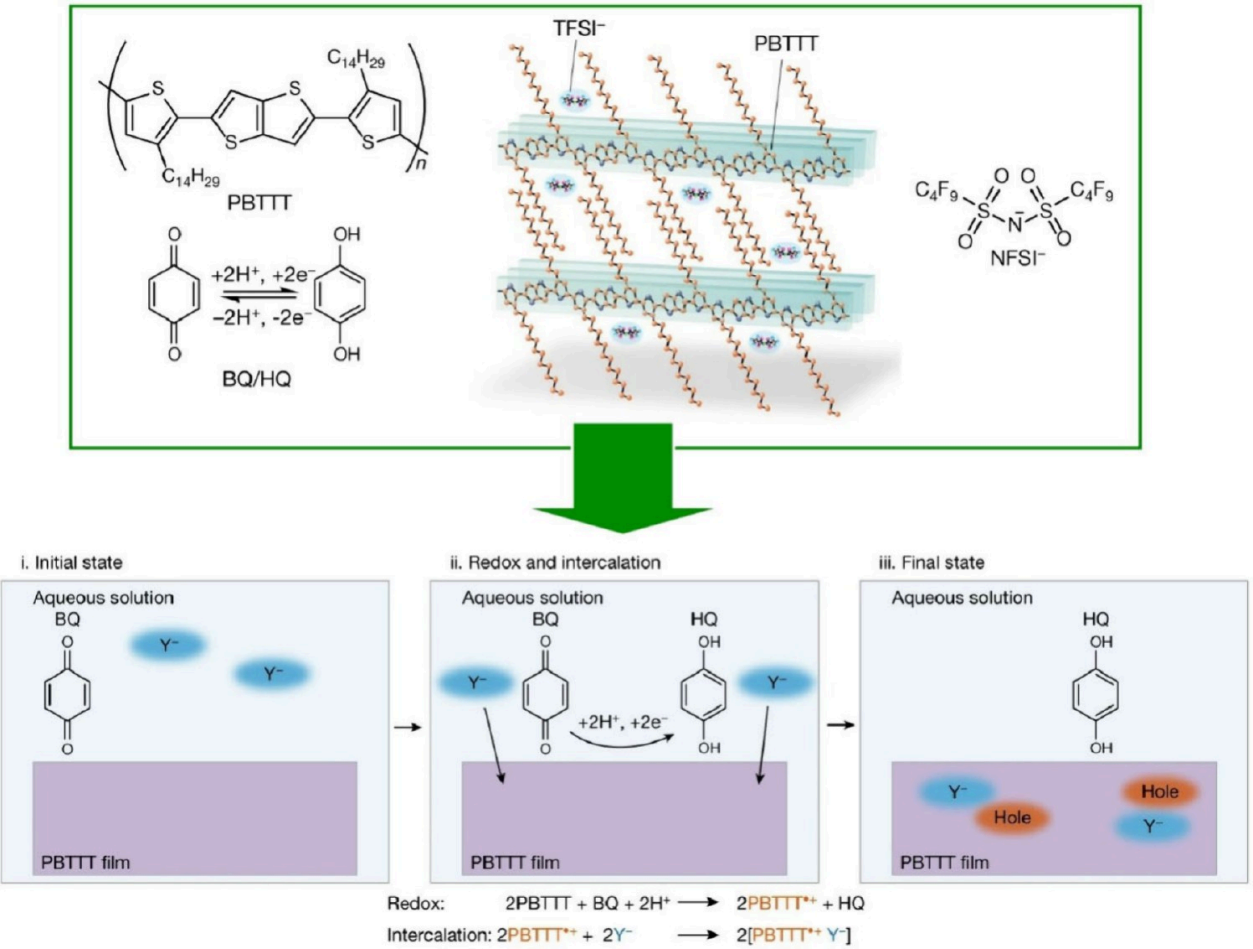
A dynamic interfacial process for combination between proton-coupled electron transfer and doping phenomenon of polymer–organic semiconductor interfaces. Reprinted with permission from ref (212). Copyright 2023 Springer-Nature.

Although it is not a macroscopic structure formation, doping control of organic semiconductor thin films can be described as an interfacial process at the molecular level. Ishii, Yamashita and co-workers have combined a process called proton-coupled electron transfer with the doping phenomenon of polymer-organic semiconductor interfaces (Figure 31).^212^ This is a new approach that combines the proton activity of the medium with the chemical doping process of organic semiconductors. When a polymer-organic semiconductor (poly[2,5-bis(3-tetradecylthiophen-2-yl)thieno[3,2-*b*]thiophen], PBTTT-C14) thin film is immersed in an aqueous solution containing a benzoquinone/hydroquinone redox pair with proton-coupled electron transfer capability, p-type doping occurs via an interfacial process. Efficient chemical doping of polymer organic semiconductors can be realized by the synergistic reaction of proton-coupled electron transfer and the insertion of hydrophobic ions. The doping level can be precisely controlled in a pH-controlled aqueous solution and the conductivity can be increased by several orders of magnitude. It is also innovative in that it can be carried out using aqueous solutions on a standard laboratory bench, whereas most conventional chemical doping methods are carried out in organic solvents in an inert atmosphere. Proton-coupled electron transfer is a process commonly used in biological systems and can contribute to bioelectronic applications. Thus, it can serve as a platform for biomolecular electronics.

**Figure 32:**
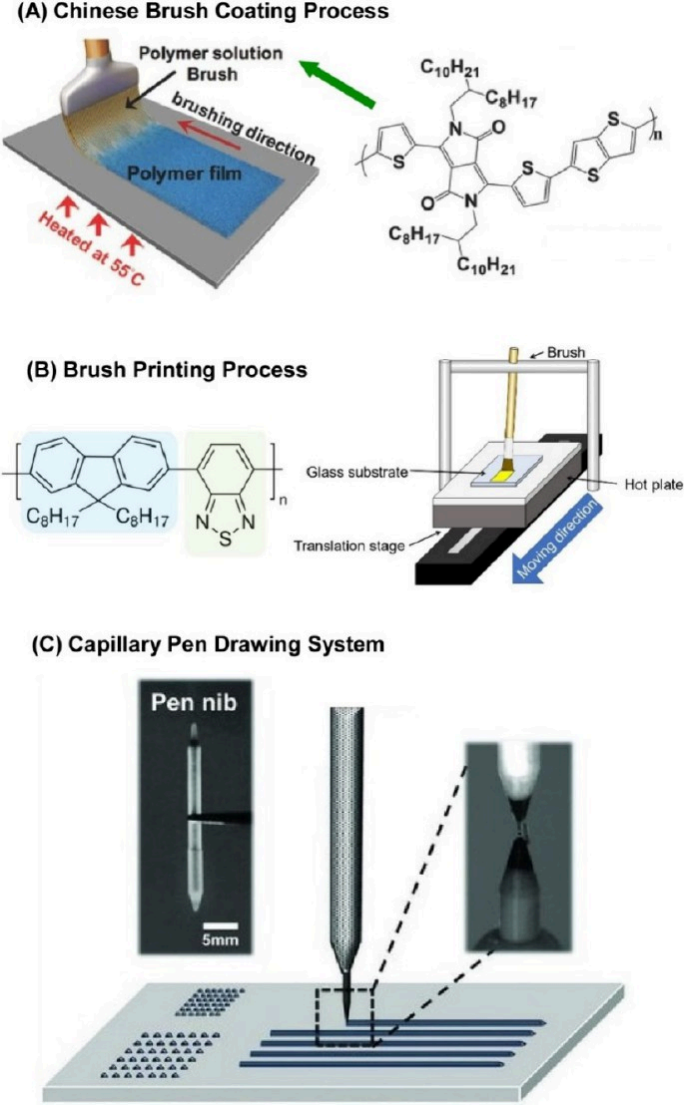
Brushing and printing orientation controls of organic semiconductor films: (A) Chinese brush coating process; (B) brush printing process; (C) capillary pen drawing system. Reprinted with permission from ref (213). Copyright 2017 Wiley-VCH. Reprinted with permission from ref (214). Copyright 2020 American Chemical Society. Reprinted with permission from ref (215). Copyright 2013 Wiley-VCH.

Macroscopic mechanical processes at interfaces have also been used to control the organization of organic semiconductor molecules such as conjugated polymers. Liu, Hsu and co-workers have shown how directional solution coating with Chinese brushes can precisely control the wetting and dewetting processes under directional stress to produce highly oriented polymer thin films (Figure 32A).^213^ With the Chinese brush coating, polymer crystallization and self-assembly of the conjugated backbone proceeds in a quasi-steady state along specific directions. Directional solution coating with Chinese brushes significantly improves the performance of conjugated polymer organic thin film transistors by more than six times compared to spin-coated films under the same experimental conditions. The better chain orientation and larger crystalline correlation length contribute to the significant improvement in mobility. This method is expected to provide an efficient approach for the simple fabrication various organic devices. Saitow and co-workers fabricated oriented films of the light-emitting semiconducting polymer poly(9,9-dioctylfluorene-*alt*-benzothiadiazole) using a brush printing method (Figure 32B).^214^ Brush printing is advantageous for the production of uniaxially oriented films. High orientation at film thicknesses below 100 nm is achieved by shear stress, fast capillary flow and flow induced chain elongation of the thin solution film on the substrate. An optimized brushing speed was determined to obtain high orientation coefficients, where non-Newtonian fluid dynamics were considered. By applying this brush-printing method to the fabrication of optoelectronic devices, organic electroluminescent devices composed of oriented light-emitting semiconducting polymer films have been demonstrated. Cho, Lee and co-workers demonstrated large-area patterning of organic electronics using a capillary pen drawing technique (Figure 32C).^215^ The tip of the pen induces capillary action in the ink. Capillary action is caused by intermolecular attraction between the liquid and solid surface in a thin tube. The tip of the pen absorbs ink from the capillaries in the tip and body of the pen and the ink flows out of the front of the tip and is deposited on the substrate. It is simple and versatile, with no limit to the shape of the pattern that can be created and tailored to different substrates. The capillary pen writing method has the potential to be extended to a variety of site-selective surface patterning structures, including for applications in organic electronics.

**Figure 33:**
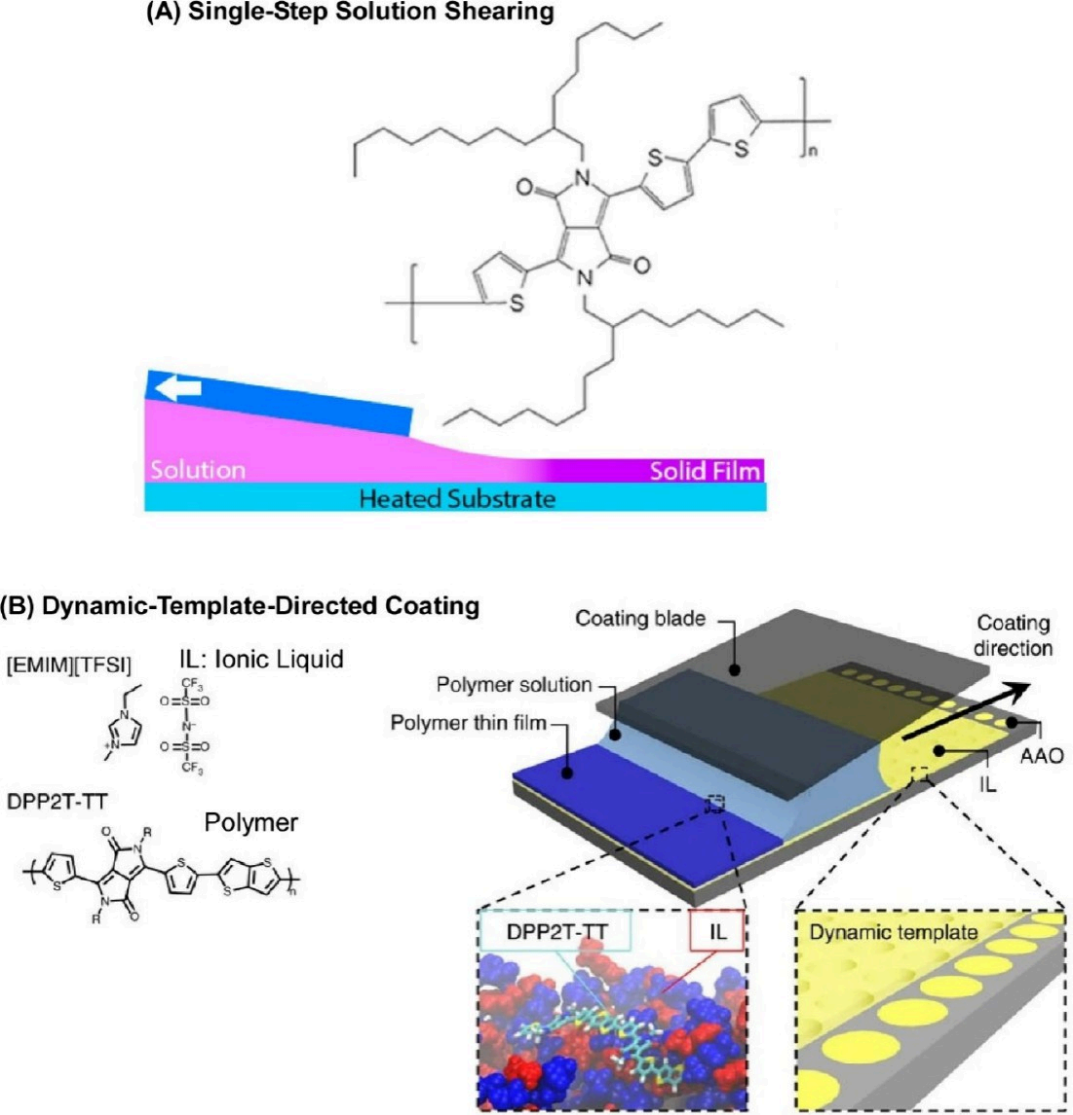
Shearing orientation controls of organic semiconductor films: (A) single-step solution shearing; (B) dynamic-template-directed coating. Reprinted with permission from ref (216). Copyright 2016 American Chemical Society. Reproduced under terms of the CC-BY license from ref (217), 2017 Springer-Nature.

The orientation of organic semiconductors typically requires complex processes. Improving charge transport through one-step solution processing and orientation is attractive. Hayoz, Bao and co-workers investigated the tuning of the in-plane orientation of a donor–acceptor semiconducting polymer, poly(diketopyrrolopyrrole-terthiophene), using solution shear as a one-step process (Figure 33A).^216^ By controlling the deposition rate, the degree of orientation of the polymer-organic semiconductor thin films was tuned by solution shear. The degree of polymer orientation is the competition between the shear orientation of the polymer chains in solution and the complicated thin film drying process. The orientation that gives the maximum dichroism can be obtained by adjusting parameters such as shear rate, temperature, and substrate treatment. This solution shear method is an example of how the polymer orientation of organic semiconductor thin films can be achieved using a one-step in situ process. A method inspired by biomineralization templates capable of surface reconstruction was also developed. Diao and co-workers proposed a method to create aligned thin films through the concept of dynamic templates that promote polymer nucleation and the subsequent assembly process (Figure 33B).^217^ The dynamic template is constructed by infiltrating the ionic liquid, 1-ethyl-3-methylimidazolium bis(trifluoromethylsulfonyl)imide, into a nanoporous matrix. Specifically, anodized aluminum oxide (AAO) with 200 nm through holes was used as the porous host. The polymer concentration lowered the nucleation barrier, promoted polymer crystallization, and mitigated the time-scale mismatch during the rapid solution coating. As a result, the polymer crystal growth is sufficiently fast to follow the receding meniscus. Unidirectional capillary flow directed the film growth along the π-π stacking direction, which is the fastest growth axis. This technique produced highly aligned and crystalline donor–acceptor polymer thin films over large areas in excess of 1 cm^2^. For field-effect transistor applications, charge transport is enhanced along both the polymer backbone and the π-π stacking direction. Furthermore, the charge transport anisotropy can be reversed by tuning the degree of orientation of the polymer backbone.

**Figure 34:**
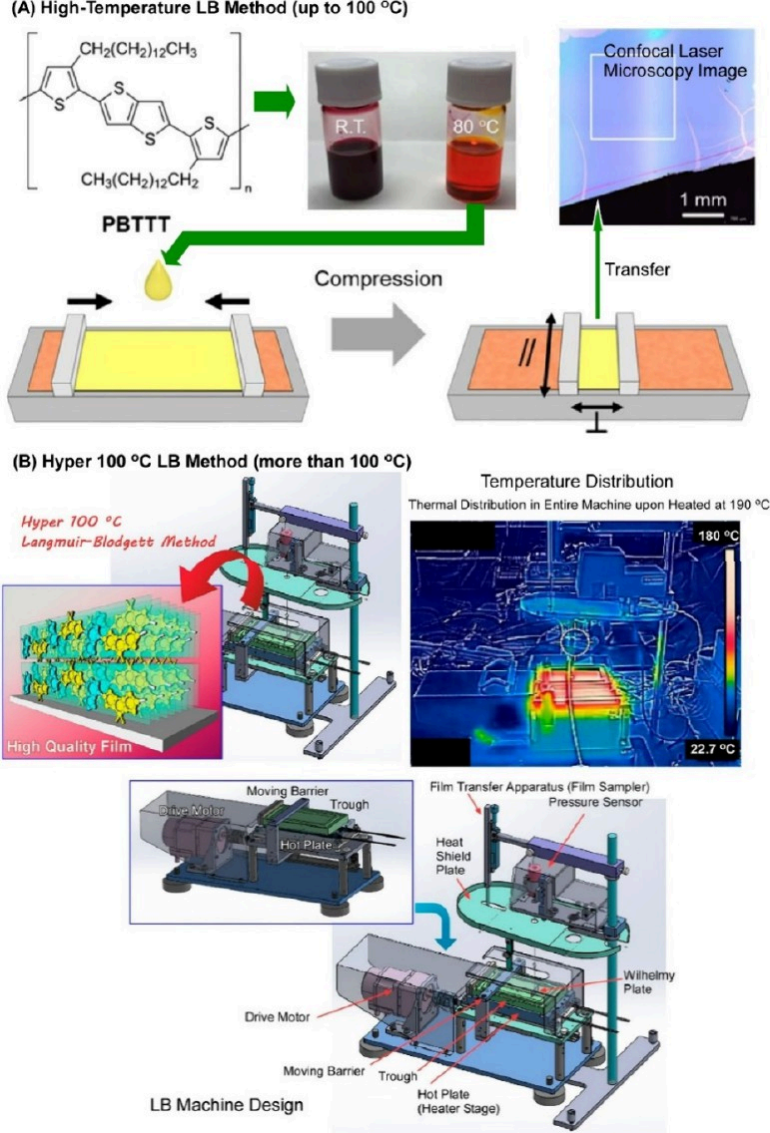
LB method at higher temperatures; (A) high-temperature LB method (up to 100 °C); (B) hyper 100 °C LB method (more than 100 °C). Reprinted with permission from ref (218). Copyright 2020 American Chemical Society. Adapted with permission from ref (219). Copyright 2021 American Chemical Society.

The LB method using a liquid surface has also made an important contribution to the production of aligned thin films of polymer semiconductors. In the LB method, an aqueous solution is generally used as the liquid phase (subphase). However, the temperature range available for the aqueous subphase is limited and not necessarily advantageous for the development of highly condensed substances. To overcome this limitation, Ito et al. proposed a method for fabricating thin films of semicrystalline polymer semiconductors by the LB method at temperatures higher than conventional temperatures using ethylene glycol, an inert and low vapor pressure liquid, as the subphase (Figure 34A).^218^ It was found that the barrier compression of the solid polymer thin film during the high-temperature LB process produces a uniaxial orientation of the polymer main chain with an average dichroic ratio of approximately 8, and at the same time, the electron transport becomes highly anisotropic. The high-temperature LB process promotes thermodynamic favorability at the air–water interface, enabling the preparation of homogeneous LB films with large-area coverage and high crystallinity. In addition, Ito et al. fabricated a new LB apparatus as a versatile device that can be adapted to high boiling point phases (Figure 34B).^219^ Using this LB machine, we extended the conventional LB method to a high-temperature range of over 100 °C. The machine has a sophisticated trough design that can uniformly heat up to approximately 200 °C, automatic film compression, and a Langmuir–Schaefer type film transfer function. The use of this machine has enabled the production of highly oriented polymer–semiconductor thin films with uniaxial orientation of the polymer backbone, which is desirable for efficient charge transport. For example, we have also succeeded in thinning a thin donor–acceptor type copolymer at a high temperature (140 °C) using an ionic liquid as a subphase. The resulting semicrystalline thin film with uniaxially oriented polymer backbone contributes significantly to the two-dimensional overlap of molecular orbitals, which facilitates charge transport. Although generally not well controlled at room temperature, the hyper 100 °C LB process using the newly fabricated LB machine successfully maintained the fluidity of the liquid phase at high temperatures and successfully fabricated uniform films with the desired orientation.

**Figure 35:**
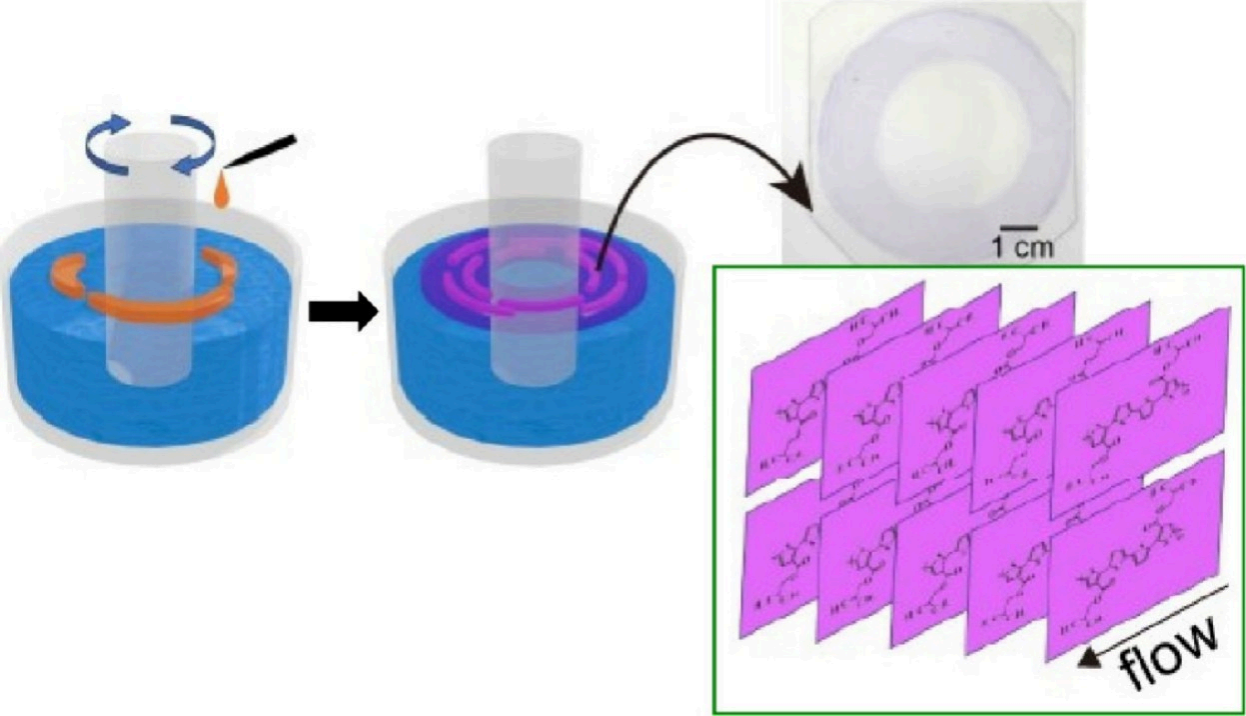
A circular flow orientation method for polymer–semiconductor thin films using glycerol as the liquid phase. Reprinted with permission from ref (220). Copyright 2025 American Chemical Society.

Orientation control of polymer semiconductors is a fundamental technique for understanding and improving the carrier transport properties. Although polymer semiconductor thin films are prepared using simple solution processes, complex convection during solvent evaporation often limits orientation control in terms of scalability and reproducibility. A system with a controlled interfacial flow can overcome these problems. To achieve this, Fujioka et al. recently developed a circular flow orientation method for polymer–semiconductor thin films using glycerol as the liquid phase (Figure 35).^220^ A polymer–semiconductor solution was dropped onto a circular flow of glycerol in a container, and a thin film was obtained at the air–liquid interface. The film obtained using the circular flow orientation method was formed in a ring shape around the cylinder. The obtained thin films had main chains oriented along the flow direction, suppressing the effect of convection during solvent evaporation. The rotation speed around the cylinder is approximately 40 mm/s. This is sufficient to promote the alignment of the polymer chains, even considering that the uniaxial alignment of the polymer chains during solution shear is less than 1 mm/s. High absorption was observed when the direction of flow and polarization of light were parallel, indicating that the main chains of the polymer semiconductor were aligned in this direction. This is also based on π-π interactions within the thin film. This method also provides a way to fabricate highly oriented, edge-on aligned ultrathin polymer–semiconductor films on air–liquid interfaces at room temperature. Field-effect transistors using the resulting polymer semiconductors exhibited four times higher mobility than spin-coated thin films under ambient conditions. This method efficiently exploits the interfacial environment as a driving force for liquid flow within the subphase. The method can also be scaled up by designing flow channels, which will contribute to a wide range of applications of aligned polymer–semiconductor thin films.

In this section, we summarize some examples of the alignment and organization of organic semiconductors by flow. Compared to semiconductor silicon, organic semiconductors have the advantages of flexibility, low-dimensionality, and lightweight, and are used devices including organic transistors, electroluminescent displays, and solar cells. Orientational control is important to obtain high performances using such low-dimensional materials. The technologies presented in the previous section are used for this purpose, but there may be more room for further developments. In particular, the liquid alignment technology described in the second half of this article has great potential. Given the ease with which macroscopic liquid flows can be controlled, the alignment of organic semiconductors by flow at interfaces will contributes to a scalable and simple manufacturing method of high-performance organic semiconductor thin films for various applications.

## 5 Summary Impression and Perspective Opinion

In this review, we have given an overview, with some examples, of (i) structural organization by natural flows, (ii) structural organization by forced flows and stresses produced by artificial instruments and devices (forced flows), and (iii) structural organization by flows in specific areas such as interfaces. Natural flow can occur anywhere, and gives considerable effects to organization of functional materials. Therefore, the study of nanostructure formation by natural flows will provide clues that are common to many structure formation processes. Considering that artificial forced flows and forces can be designed in diverse ways, the creation of structures by forced flows is an approach with great ripple effects. Although we have not gone into detail in this review, the microfluidic systems^221−223^ offer an attractive method in this context. Using more rationally integrated instruments and devices could be a powerful method for creating functional materials with desired structures. The dynamic system other than mechanical flows such as such as the electrophoretic method^224^ has to be well included although it was not mentioned in the previous sections. In addition, more considerations on common applications methods on standard materials such as PEDOT:PSS-based conducting polymers^225−227^ have to be done for wider generations of these methodologies. One of the important keys is to establish a theory of coupling between fluid dynamics and supramolecular chemistry, such as the out-of-equilibrium self-assembly. Interfaces are valuable sites at which large forces can be coupled with molecular phenomena. They have great potential for controlling the structure of tiny molecules using macroscopic forces. In addition, the fabrication of material structures on liquid surfaces is expected to be easier. Large-scale production is also possible. Several examples of organic semiconductors have been provided, where there seems to be room for improvements to establish a manufacturing method with molecular precision. This direction with integration of nanoscale phenomena into large-scale systems and applications would create the sustainability and environmental impacts with flow-assisted nanoarchitectonics.

Based on these facts and summaries, we will consider what is needed for future research as challenges in integration of nanoscale phenomena and macroscopic actions with flow-assisted methods. These research projects do not end with the narrow goal of creating array structures using flow. There is a very large hidden goal of rationally integrating nanotechnology into material production and processing techniques that humans have long used to create functional materials. It is essential that this is brought together as a system rather than as a separate issue. To achieve this, it is necessary to build a theoretical framework that combines several fields. Guidelines should be established that incorporate the elements of fluid mechanics, supramolecular chemistry, thermodynamics, interfacial science and more. This can also help elucidate dynamic scientific phenomena such as processes in living organisms. Development from a more engineering perspective is required. There are many possibilities for engineering applications of material organization by flow, from microfabrication by combining microfluidics to mass production using large-area water tank interfaces. It may not be easy to unify these varieties using a single framework. Rather, harnessing the power of artificial intelligence to find optimal solutions might be an effective approach in both engineering and industry. The use of machine learning to create functional materials is also becoming more common.^228−232^ There is also a need to integrate the fields of materials informatics and nanoarchitectonics.^231,228^ It is expected that this will become possible through the accumulation of data on the strength and quality of the flow, the material used, the shape of the equipment used, the use of the field such as the interface, and the structure such as the orientation of the material to be created. Especially, establishments of common models based on these factors using artificial intelligence approaches would give important guidelines for materials production upon flow-assisted nanoarchitectonics. A dynamic method of creating organizational structures that contribute to industrial applications can be established with the aid of artificial intelligence.

In this review, we provide an overview of the control of material structure, mainly by flow, under the title of dynamic flow-assisted nanoarchitectonics. This is not a narrow topic of simply controlling structures by flow, but rather a topic that will provide an approach to the general and highly important goal of incorporating nanotechnology into the fabrication of macroscopic conventional functional materials. What awaits us in the future is the meaningful and effective use of nanotechnology and nanoarchitectonics in materials science. The consideration of dynamic flow-assisted nanoarchitectonics would become a good opening gate for this future goal.
